# Exploring *Pseudomonas syringae* pv. *tomato* biofilm‐like aggregate formation in susceptible and PTI‐responding *Arabidopsis thaliana*

**DOI:** 10.1111/mpp.13403

**Published:** 2023-11-21

**Authors:** Wantao N. Xiao, Garrett M. Nunn, Angela B. Fufeng, Natalie Belu, Rowan K. Brookman, Abdul Halim, Evan C. Krysmanski, Robin K. Cameron

**Affiliations:** ^1^ Department of Biology McMaster University Hamilton Ontario Canada

**Keywords:** alginate, biofilm‐like aggregates, leaf intercellular space, PAMP‐triggered immunity, salicylic acid

## Abstract

Bacterial biofilm‐like aggregates have been observed in plants, but their role in pathogenicity is underinvestigated. In the present study, we observed that extracellular DNA and polysaccharides colocalized with green fluorescent protein (GFP)‐expressing *Pseudomonas syringae* pv. *tomato* (Pst) aggregates in *Arabidopsis* leaves, suggesting that Pst aggregates are biofilms. GFP‐expressing Pst, Pst Δ*algU* Δ*mucAB* (Pst *algU* mutant), and Pst Δ*algD* Δ*algU* Δ*mucAB* (Pst *algU algD* mutant) were examined to explore the roles of (1) alginate, a potential biofilm component; (2) Pst AlgU, thought to regulate alginate biosynthesis and some type III secretion system effector genes; and (3) intercellular salicylic acid (SA) accumulation during pathogen‐associated molecular pattern‐triggered immunity (PTI). Pst formed extensive aggregates in susceptible plants, whereas aggregate numbers and size were reduced in Pst *algU* and Pst *algD algU* mutants, and both multiplied poorly in planta, suggesting that aggregate formation contributes to Pst success in planta. However, in SA‐deficient *sid2‐2* plants, Pst *algD algU* mutant multiplication and aggregate formation were partially restored, suggesting plant‐produced SA contributes to suppression of Pst aggregate formation. Pst *algD algU* mutants formed fewer and smaller aggregates than Pst *algU* mutants, suggesting both AlgU and AlgD contribute to Pst aggregate formation. Col‐0 plants accumulated low levels of SA in response to Pst and both mutants (Pst *algU* and Pst *algD algU*), suggesting the regulatory functions of AlgU are not involved in suppressing SA‐mediated plant defence. Plant PTI was associated with highly reduced Pst aggregate formation and accumulation of intercellular SA in flg22‐induced PTI‐responding wild‐type Col‐0, but not in PTI‐incompetent *fls2*, suggesting intercellular SA accumulation by *Arabidopsis* contributes to suppression of Pst biofilm‐like aggregate formation during PTI.

## Graphical abstract

PTI‐responding leaves accumulate intercellular SA and few GFP‐expressing *Pseudomonas* aggregates, whereas susceptible leaves do not accumulate intercellular SA and have many large GFP‐expressing *Pseudomonas* aggregates.

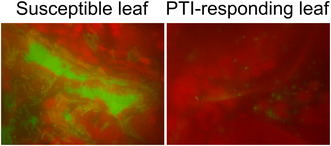

## 1 INTRODUCTION

A biofilm is defined as a group of microbial cells associated with a surface and enclosed in a matrix of extracellular polymeric material (Donlan, 2002). Biofilm formation by the human opportunistic pathogen *Pseudomonas aeruginosa* has been studied extensively because biofilm formation is required for infection of cystic fibrosis patients (Hoiby et al., 2010; Rasamiravaka et al., 2015). It is believed that biofilms physically prevent host immune cells from binding to *P. aeruginosa* within the biofilm, allowing *P. aeruginosa* to evade host immune defences (Moser et al., 2021). In contrast, biofilm formation during plant–microbe interactions has received less attention (Heredia‐Ponce et al., 2021). Plant‐associated bacteria have been observed to form aggregates (tightly packed bacterial cells) on plant cell surfaces and in xylem vessels. For example, *Pseudomonas fluorescens* forms aggregates on rice and wheat root surfaces (Couillerot et al., 2009). *Sinorhizobium meliloti* forms bacterial aggregates in alfalfa nodules. *S. meliloti* mutants with reduced extracellular matrix production display a reduced ability to induce alfalfa nodulation (Fujishige et al., 2006). *Xylella fastidiosa*, a pathogen of grapevine, forms bacterial aggregates on inner xylem vessel walls to cause Pierce's disease (Marques et al., 2002). The bacterial pathogen *Erwinia amylovora* forms aggregates on xylem vessel walls during infection of apple and pear tree leaves (Koczan et al., 2009). *E. amylovora* aggregates contribute to successful infection, as demonstrated by a mutant strain unable to produce the extracellular polysaccharide amylovoran or form biofilm‐like aggregates in apple leaves (Koczan et al., 2009). Recent evidence suggests that aggregate formation and secretion of phytotoxins by *Pseudomonas savastanoi* pv. *phaseolicola* occur as bacterial countermeasures to bean plant immune responses (Cooper et al., 2021). Because aggregate formation is part of the biofilm formation process, these studies support the idea that biofilm formation contributes to pathogenicity and virulence of bacterial plant pathogens. Pathogenicity is defined as the ability to cause disease in a host species, a qualitative measure, and virulence is defined as the degree of disease, a quantitative measure (Sacristan et al., 2021).

In the *Arabidopsis thaliana*–*Pseudomonas syringae* pathosystem, *P. syringae* was observed to form aggregated cells in susceptible plants in a number of electron and epifluorescence microscopy studies (Badel et al., 2002; Boureau et al., 2002; Varvaro et al., 1993; Whalen et al., 1991). It is unknown if these aggregates are biofilms and if biofilm formation contributes to the pathogenicity and success of *P. syringae* in planta. In a scanning electron microscopy study, *P. syringae* aggregates were cultured in vitro and observed to be encased in an extracellular matrix (Farias & Olmedilla, 2019). In vitro studies demonstrated that the extracellular matrix of biofilms contains various components, including polysaccharides, proteins, extracellular DNA (eDNA), and lipids (reviewed in Flemming & Wingender, 2010). A study by Buell et al. (2003) revealed that the genome of the tomato and *Arabidopsis* pathogen *P. syringae* pv. *tomato* (Pst) contains extracellular polysaccharide biosynthesis genes that are orthologous to genes in *P. aeruginosa*. The polysaccharide alginate is thought to be an important matrix component because it is produced in human chronic pulmonary infections, but not in other environments (e.g., soil), suggesting that alginate production is important for *P. aeruginosa* pathogenicity (Hoiby et al., 2010; Muhammadi & Ahmed, 2007). Additionally, alginate‐containing biofilm matrices are thought to interfere with host immune responses to protect *P. aeruginosa* from hostile host environments (Mishra et al., 2015). Given that the Pst genome contains orthologues of *P. aeruginosa* alginate biosynthesis genes, alginate production may play an important role in Pst aggregate formation in planta.

Pst contains the genomic loci for synthesis of the polysaccharides cellulose, levan, polysaccharide single locus, and alginate (Buell et al., 2003). The ability to produce cellulose may not be necessary for bacterial success as similar levels of multiplication were observed for cellulose synthase mutants and wild‐type Pst in tomato (Prada‐Ramírez et al., 2016). The ability to suppress plant defence was also intact in cellulose synthase mutants of Pst, suggesting that polysaccharide production and biofilm formation are not necessary when Pst is able to suppress plant defence. In support of this idea, little cellulose synthase expression was detected when virulent Pst caused disease in *Arabidopsis*, whereas cellulose synthase was highly expressed by Pst *avrRpt2* during effector‐triggered immunity (ETI) (Nobori et al., 2018).

Alginate may also be a component of the Pst biofilm matrix as alginate‐deficient Pst Δ*algD* mutants cause typical necrotic lesions, but with less chlorosis on tomato leaves compared to wild‐type Pst (Ishiga et al., 2018), suggesting that alginate biosynthesis may contribute to the production of disease symptoms. Alginate is a copolymer of β‐(1,4)‐mannuronic and α‐(1,4)‐guluronic acid (Grasdalen, 1983) and its biosynthesis has been studied in *P. aeruginosa* (Rasamiravaka et al., 2015). AlgD encodes GDP‐d‐mannose dehydrogenase, which catalyses the oxidation of GDP‐d‐mannose to GDP‐d‐mannuronic acid, the final step in alginate precursor production, followed by polymerization (Fakhr et al., 1999). The alginate production mutant Pst Δ*algD* multiplied like wild‐type Pst in tomato (Markel et al., 2016). However, an approximately threefold reduction in bacterial levels was observed in *Arabidopsis* seedlings infected with Pst Δ*algD* compared to those infected with wild‐type Pst (Ishiga et al., 2018). It appears that alginate production may be important during some Pst infections of the leaf intercellular space.

Alginate biosynthesis is controlled in *P. aeruginosa* by a number of regulators including *AlgU* and *MucAB* (Hay et al., 2013). These regulators are also found in Pst (Buell et al., 2003). Many biofilm‐forming *P. aeruginosa* isolates have mutations that abolish MucA or MucB anti‐sigma factor function, resulting in AlgU activity even under nonbiofilm‐inducing conditions (Martin et al., 1993; Mathee et al., 1997; Schurr et al., 1996; Xie et al., 1996). *AlgU* encodes a sigma factor that regulates alginate biosynthesis genes including *AlgD* and the type III secretion system (T3SS) gene *HrpL* (Ishiga et al., 2018). *HrpL* expression was reduced in *Arabidopsis* seedlings flood‐inoculated with Pst Δ*algU* (Ishiga et al., 2018). HrpL is an alternative RNA polymerase sigma factor involved in regulating expression of some T3SS effectors during Pst infection (Fouts et al., 2002). Current evidence suggests that AlgU contributes to regulating T3SS‐associated virulence functions, alginate production, and flagellin expression during Pst infection of *Arabidopsis* (Bao et al., 2020; Ishiga et al., 2018; Markel et al., 2018).

In several studies, the *Arabidopsis* age‐related resistance (ARR) response in mature plants was associated with salicylic acid (SA)‐dependent suppression of both Pst multiplication (Cameron & Zaton, 2004; Kus et al., 2002) and biofilm‐like aggregate formation in leaf intercellular spaces compared to young susceptible plants (Wilson et al., 2017). Several in vitro studies demonstrated that SA exhibited antimicrobial activity against various phytopathogens (Amborabé et al., 2002; Brown et al., 2007; Cameron & Zaton, 2004; Georgiou et al., 2000; Ortiz‐Martín et al., 2010; Prithiviraj et al., 2005; Wilson et al., 2017). Additionally, SA concentrations of 5 to 100 μM reduced Pst biofilm formation in vitro (Wilson et al., 2017) and similar levels of SA (40 to 100 μM) accumulated in intercellular washing fluids (IWFs) collected from mature ARR‐responding leaves, but not in IWFs collected from young ARR‐incompetent leaves inoculated with Pst (Cameron & Zaton, 2004; Kus et al., 2002; Wilson et al., 2017). These experiments support the idea that intercellular SA acts as an important antimicrobial and antibiofilm agent during ARR in mature leaves.

Less is known about the antimicrobial role of SA during pathogen‐associated molecular pattern (PAMP)‐triggered immunity (PTI) or ETI in young plants. During ETI, resistance receptors perceive pathogen‐produced effector proteins (directly or indirectly) to initiate ETI signalling and defence (Mine et al., 2018). ETI includes intercellular SA accumulation; however, bacterial biofilm‐like aggregate formation was not investigated (Carviel et al., 2014). Additionally, intercellular SA accumulation and bacterial biofilm‐like aggregate formation during PTI have not been investigated. A study of the interaction between the bacterial flagellin peptide flg22 and the plant pattern recognition receptor FLAGELLIN SENSITIVE 2 (FLS2) has contributed to our understanding of PTI (Jelenska et al., 2017). *Arabidopsis* FLS2 binds directly to flg22 to initiate PTI, which results in resistance to Pst (Zipfel et al., 2004). PTI includes cell wall strengthening and production of antimicrobial chemicals and pathogenesis‐related proteins at infection sites (Bigeard et al., 2015), and SA is thought to act as an intracellular signal during PTI (Mine et al., 2018).

In the present study, both sides of the Pst–*Arabidopsis* interaction were examined using epifluorescence microscopy to visualize green fluorescent protein (GFP)‐expressing wild‐type Pst and alginate mutants in leaf intercellular spaces. The ability of Pst to form many large biofilm‐like aggregates was correlated with pathogenicity and bacterial multiplication in planta. In addition, Pst biofilm‐like aggregates in leaf intercellular spaces colocalized with eDNA and carbohydrates, providing support that Pst forms biofilms during infection of *Arabidopsis*. On the plant side of the interaction, the effect of the PTI response on Pst biofilm‐like aggregate formation was examined. Like the ARR response, SA accumulated in the intercellular space during PTI and was associated with reduced bacterial biofilm‐like aggregate formation.

## 2 RESULTS

### 2.1 Bacterial aggregate formation is associated with successful infection by Pst

**FIGURE 1:**
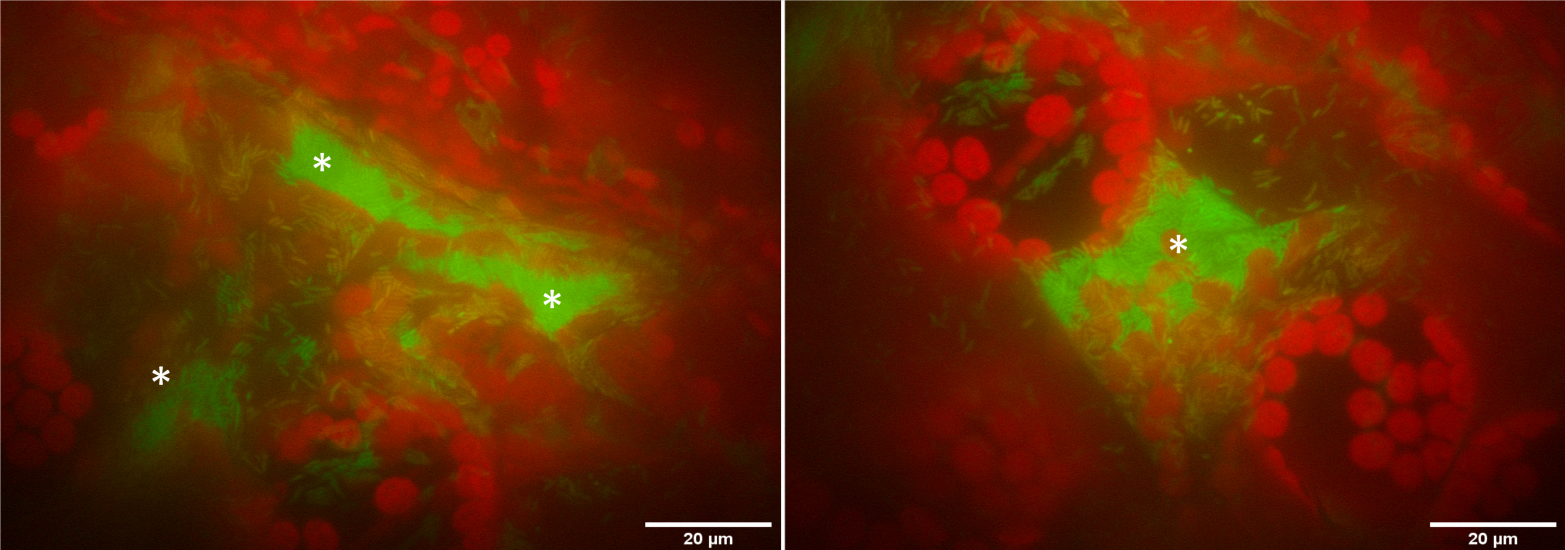
Visualization of green fluorescent protein (GFP)‐expressing *Pseudomonas syringae* pv. *tomato* (Pst) in susceptible leaves. Leaves and bacteria were viewed at 1000× magnification under epifluorescence in plants inoculated with Pst (10^6^ cfu/leaf disc [ld]) at 2 days post‐inoculation. Asterisks mark examples of aggregated bacteria. The results of one experiment of many performed over two years are shown (Table S4).

Given that biofilm‐like aggregates were observed in young plants inoculated with Pst in *Arabidopsis* (Wilson et al., 2017), we hypothesized that the ability to form biofilm‐like aggregates is associated with Pst success and virulence. Pst success was determined by measuring in planta bacterial levels and a GFP‐expressing Pst strain was used to monitor biofilm‐like aggregate formation in leaf intercellular spaces. As expected, leaves inoculated with virulent GFP‐expressing Pst supported high levels of bacterial multiplication 3 days post‐inoculation (dpi, c.10^7^ cfu/leaf disc [ld]). Using epifluorescence microscopy, GFP‐expressing Pst cells were classified as aggregates (biofilm‐like), defined as immobile and tightly grouped cells, or planktonic cells, defined as individual free‐swimming cells. Pst aggregates and planktonic cells were observed in leaf intercellular spaces between plant cells delineated by red autofluorescent chloroplasts at 2 dpi (Figure 1). Pst aggregate numbers and sizes were also determined in several experiments discussed below. Over eight experiments, high in planta Pst multiplication was shown to be significantly correlated with high Pst aggregate numbers (Figure S6).

### 2.2 Visualization of the Pst extracellular matrix

**FIGURE 2:**
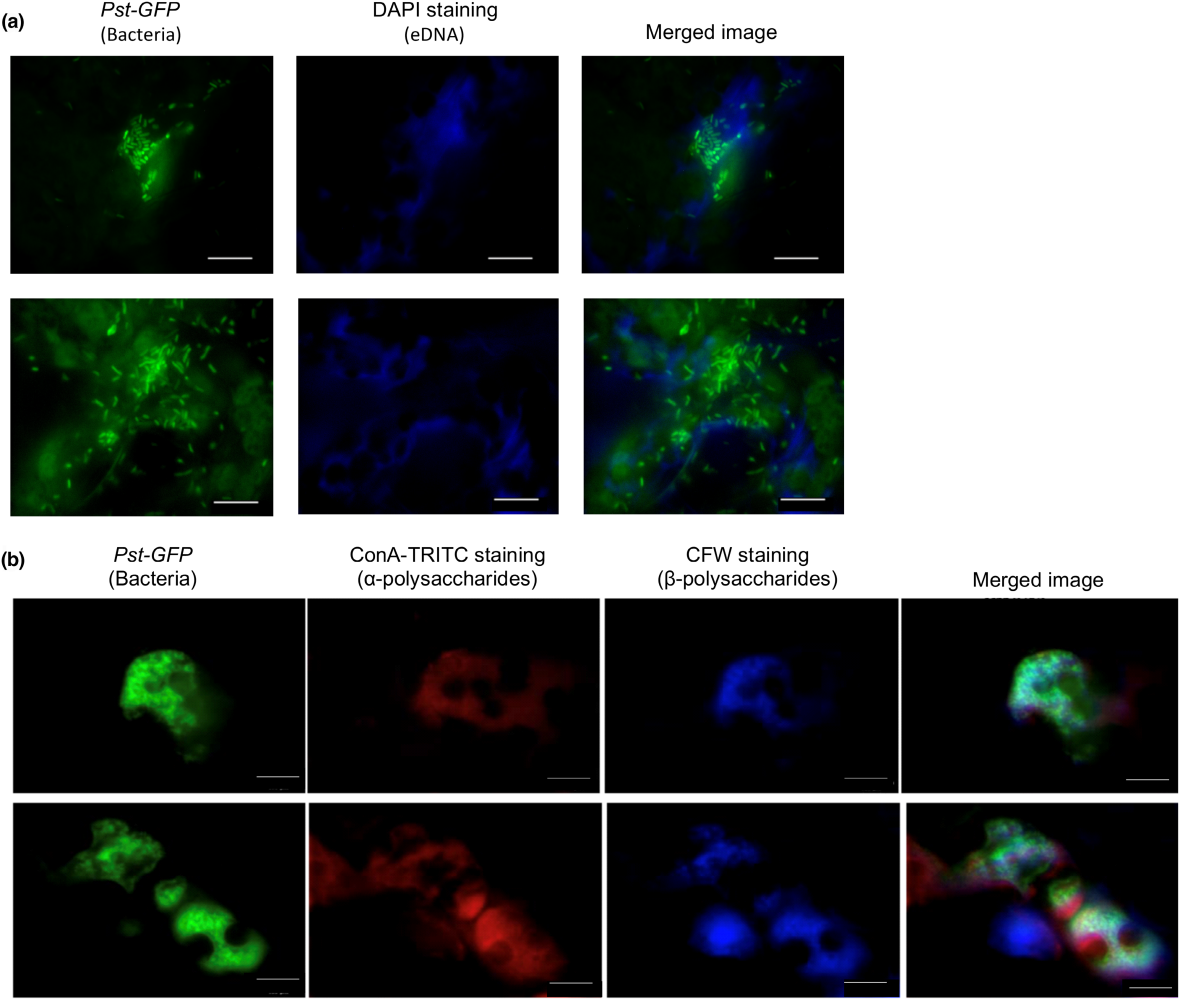
Visualization of the *Pseudomonas syringae* pv. *tomato* (Pst) extracellular matrix. The epidermis was peeled from leaves inoculated with Pst‐GFP at 48 h post‐inoculation and then stained with (a) DAPI to visualize extracellular DNA (UV filter, 435–585 nm) or (b) ConA‐TRITC to visualize α‐polysaccharides (Cy5 filter, 663–738 nm) and calcofluor white (CFW) to visualize β‐polysaccharides (UV filter, 435–485 nm) using a fluorescence microscope. Scale bar 20 μm. This experiment was performed two additional times with similar results observed.

The extracellular matrix of bacterial biofilms contains bacterial proteins, lipids, and eDNA (Mann & Wozniak, 2012). To obtain evidence that Pst aggregates are biofilms, stains for components of the extracellular matrix were applied to infected leaves and fluorescence microscopy was used to visualize bacterial aggregates and matrix components. The epidermis of leaves inoculated with GFP‐expressing Pst were peeled to expose inner leaf cells and intercellular spaces and then incubated in 4′,6‐diamidino‐2‐phenylindole (DAPI) staining solution to observe eDNA. GFP‐expressing Pst aggregates were observed using a GFP filter (510 nm) and eDNA was observed using a UV filter (435–485 nm) to detect blue DAPI signals. DAPI signals were observed to surround and overlap with Pst‐GFP aggregates in the merged images in Figure 2a. Because calcofluor white emits signals with a similar wavelength as DAPI, other leaves were peeled and stained with ConA‐TRITC and calcofluor white to visualize α‐ and β‐polysaccharides, respectively. Extracellular α‐ and β‐polysaccharides were detected as red signals (Cy5 filter, 663–738 nm) and blue signals (UV filter, 435–485 nm), respectively (Figure 2). These signals overlapped and surrounded GFP‐expressing Pst aggregates in the merged images, suggesting that GFP‐expressing Pst were embedded in a matrix of polysaccharides and eDNA as part of an in planta biofilm (Figure 2b).

To quantitatively determine if GFP‐expressing bacteria colocalized with ConA‐TRITC signals or calcofluor white signals, GFP fluorescence images were superimposed with the corresponding ConA‐TRITC‐ or calcofluor white‐stained images. ImageJ (Abramoff et al., 2004) with the JACoP plug‐in (Bolte & Cordelières, 2006) was used to calculate colocalization of GFP signals with either ConA‐TRITC or calcofluor white signals. Pixel‐matched signal intensity from each channel was used to calculate the Pearson correlation coefficient, Li's intensity quotient (Li et al., 2004), and Mander's split co‐occurrence (Manders et al., 1993). Nine aggregates were stained, imaged, and analysed using all three methods (see Figure S1 for data and a detailed analysis). These analyses demonstrated that ConA‐TRITC and calcofluor white colocalized with GFP‐expressing Pst aggregates, suggesting aggregates of Pst are surrounded by an extracellular matrix of α‐ and β‐polysaccharides.

### 2.3 Pst Δ*algD* formed fewer aggregates, but multiplied as well as Pst in SA‐producing plants

Alginate is an important component of the extracellular matrix of *P. aeruginosa* biofilms (Rasamiravaka et al., 2015), and Pst encodes alginate biosynthesis genes (Buell et al., 2003); therefore, the contribution of alginate to bacterial aggregate formation was investigated. *Arabidopsis* leaves were inoculated with virulent Pst‐GFP or Pst‐GFP Δ*algD*, an alginate biosynthesis mutant (Markel et al., 2016). The effect of plant‐produced SA on wild‐type Pst and Pst Δ*algD* was also investigated by comparing the multiplication and aggregate formation (numbers and size) of GFP‐expressing virulent Pst and Pst Δ*algD* in Col‐0 and SA‐deficient *sid2‐2* plants. Both Col‐0 and *sid2‐2* plants inoculated with wild‐type Pst and Pst Δ*algD* supported similar levels of bacterial multiplication (Figure S2a). In addition, Pst and Pst Δ*algD* grew similarly well over 72 h when cultured in *hrp*‐inducing medium that mimics the leaf intercellular environment (Figure S3a). These data suggest that the ability to produce alginate is not important for Pst multiplication in *Arabidopsis*.

During this experiment (Figures S2 and S3), quantitative aggregate size data were obtained by determining the area of aggregates present in 20 fields of view per treatment group using ImageJ. The aggregates were categorized as tiny (<100 μm^2^), small (100–199 μm^2^), medium (200–299 μm^2^), or large (>300 mμm^2^) (Figure S2c). Wild‐type Pst formed 30 large aggregates (32 total aggregates) in Col‐0 plants, whereas Pst Δ*algD* formed only six large aggregates, as well as six medium, two small, and four tiny aggregates (18 in total) (Figure S2b), indicating that in the absence of alginate in the Pst Δ*algD* mutant, aggregate numbers and size were reduced. In SA‐deficient *sid2‐2* leaves, Pst Δ*algD* formed many large aggregates (21 of 24), similar to wild‐type Pst (Figure S2b), suggesting that plant‐produced SA negatively impacts Pst aggregate formation. These data suggest that alginate is important for Pst aggregate formation when bacterial cells are exposed to plant‐produced SA.

### 2.4 Pst Δ*algD* Δ*algU* Δ*mucAB* multiplied poorly and formed fewer aggregates compared to Pst

**FIGURE 3:**
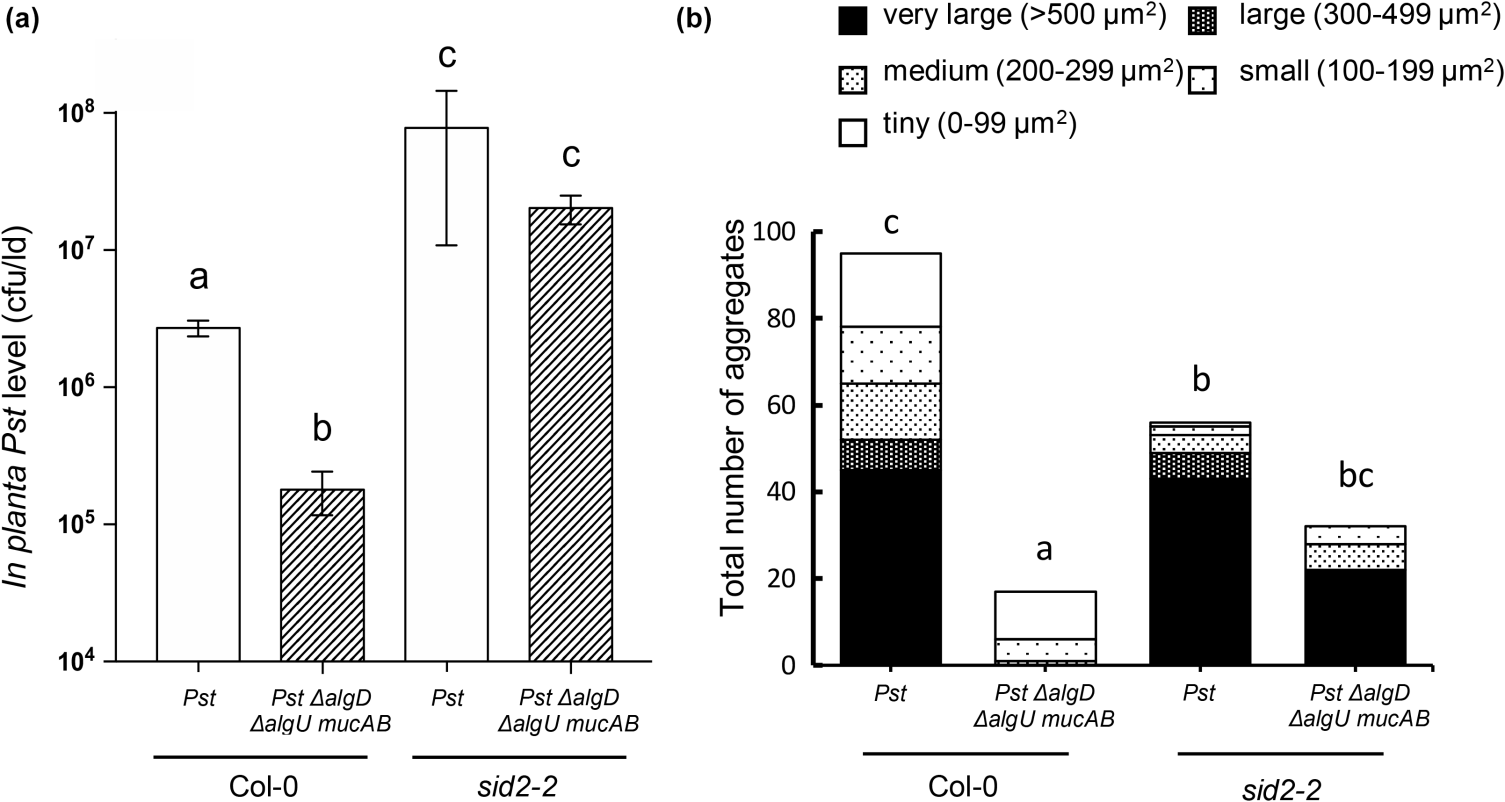
Growth and aggregate formation of *Pseudomonas syringae* pv. *tomato* (Pst) and Pst Δ*algD* Δ*algU* Δ*mucAB* in wild‐type Col‐0 and *sid2‐2*. Leaves were inoculated with green fluorescent protein (GFP)‐expressing wild‐type Pst or GFP‐expressing Pst Δ*algD* Δ*algU* Δ*mucAB* (10^6^ cfu/mL). (a) In planta bacterial quantification of both strains in Col‐0 and *sid2‐2* (salicylic acid biosynthesis mutant) at 48 h post‐inoculation (hpi). The *y* axis is cfu/leaf disc (ld) in log scale. Different letters indicate significant differences as determined using two‐way analysis of variance followed by Tukey's honestly significant difference test (*p* < 0.05). (b) Aggregate formation was monitored by categorizing each microscopic field of view (40 fields of view per treatment) containing bacterial aggregates of different sizes at 48 hpi. Different letters indicate significant differences in aggregate size distribution among groups as determined using the Kruskal–Wallis test (*p* < 0.05). The experiment in panels (a) and (b) was performed two additional times with similar results.

The Pst Δ*algD* mutant multiplied as well as Pst in planta, perhaps because it possesses an intact T3SS to deliver effectors into plant cells, some of which are known to suppress SA‐mediated defence (Bauters et al., 2021). Therefore, the ability to form aggregates was examined in a Pst mutant (Pst Δ*algD* Δ*algU* Δ*mucAB*) that cannot produce alginate and was shown to have reduced T3SS effector gene expression due to a mutation in the AlgU sigma factor (Markel et al., 2016). A GFP‐expressing Pst Δ*algD* Δ*algU* Δ*mucAB* strain was created and compared to wild‐type Pst‐GFP in terms of multiplication and aggregate formation in leaves. Col‐0 plants inoculated with Pst Δ*algD* Δ*algU* Δ*mucAB* supported 15‐fold lower bacterial levels than plants inoculated with wild‐type Pst (Figure 3a). This is consistent with the in vitro observation that Pst Δ*algD* Δ*algU* Δ*mucAB* displayed reduced growth compared to wild‐type Pst when cultured in leaf intercellular space‐mimicking medium over 72 h (Figure S3b). In terms of the mutant's ability to form aggregates in wild‐type Col‐0 leaves, far fewer and smaller aggregates (18 in total) were observed compared to wild‐type Pst, which formed 95 aggregates in total, of which 45 were very large (Figure 3b). Therefore, Pst Δ*algD* Δ*algU* Δ*mucAB* formed far fewer aggregates and multiplied poorly compared to fully virulent wild‐type Pst, suggesting that Pst Δ*algD* Δ*algU* Δ*mucAB*'s lack of AlgU regulation contributed to making these alginate‐lacking bacterial cells more susceptible to the plant defence response.

### 2.5 Effect of plant‐produced SA on Pst Δ*algD* Δ*algU* Δ*mucAB* aggregate formation and size

A study by Wilson et al. (2017) provided in vitro and in vivo evidence that supports the idea that plant‐produced intercellular SA acts as an antibiofilm agent during the ARR response in mature plants (Wilson et al., 2017). To begin to investigate if intercellular SA also acts as an antibiofilm agent in young plants, the effect of SA‐mediated resistance on Pst and Pst Δ*algD* Δ*algU* Δ*mucAB* mutant multiplication and aggregate formation was compared in SA‐deficient *sid2‐2* and Col‐0 plants. Pst Δ*algD* Δ*algU* Δ*mucAB* grew to higher levels in *sid2‐2* compared to Col‐0 and reached similar bacterial levels as wild‐type Pst in *sid2‐2* plants (Figure 3a). Moreover, intact SA‐mediated defence in Col‐0 was more effective in reducing Pst Δ*algD* Δ*algU* Δ*mucAB* levels compared to wild‐type Pst (Figure 3a). Therefore, it is possible that wild‐type plant‐produced SA reduced Pst Δ*algD* Δ*algU* Δ*mucAB* multiplication because this mutant cannot make alginate and is also less able to suppress SA‐mediated plant defence due the *algU* mutation that results in reduced T3SS effector gene expression (Markel et al., 2016).

To investigate the effect of plant‐produced SA on aggregate formation, the numbers and size of aggregates in 40 fields of view were compared in Col‐0 and *sid2‐2*. Pst Δ*algD* Δ*algU* Δ*mucAB* formed almost double the number of aggregates (32 vs. 17) along with 22 very large aggregates in *sid2‐2* compared to 0 very large aggregates in Col‐0 leaves (Figure 3b). However, in Col‐0 leaves inoculated with Pst, 17 fields of view contained one very large aggregate (ranging from 725 to 8270 μm^2^) and many fields of view contained two to seven aggregates, and 50% of these aggregates (39 out of 78 aggregates) were larger in size (253 to 8880 μm^2^), whereas all Pst Δ*algD* Δ*algU* Δ*mucAB* aggregates were less than 245 μm^2^ in size in Col‐0 leaves. Although one field of view contained seven Pst Δ*algD* Δ*algU* Δ*mucAB* aggregates in Col‐0 leaves, these aggregates consisted of one small (156 μm^2^) and six tiny (1–99 mμm^2^) aggregates. These results suggest that wild‐type levels of in planta bacterial multiplication are associated with the ability of Pst to form large biofilm‐like aggregates.

It was surprising to observe that the total number of aggregates was lower for Pst in *sid2‐2* (56) versus wild‐type Col‐0 (95) (Figure 3c). Upon further examination, Pst formed one field of view‐filling aggregate in 25 fields of view out of 40 in *sid2‐2* leaves, whereas Pst formed one field of view‐filling aggregate in 16 fields of view out of 40 in Col‐0 leaves (Figure 3c). This may account for the low Pst aggregate numbers in *sid2‐2* and suggests that Pst was more successful in *sid2‐2* compared to Col‐0. This provides further support for the idea that although Pst suppresses SA‐mediated plant defence (Bauters et al., 2021), the pathogen still benefits from growing in *sid2‐2* plants that produce little SA. In SA‐deficient *sid2‐2*, Pst Δ*algD* Δ*algU* Δ*mucAB* formed more large aggregates (>300 mμm^2^) than in Col‐0 (22 vs. 0) and these large aggregates were similar in size to wild‐type Pst aggregates observed in *sid2‐2*, suggesting that plant‐produced SA affects the size and number of biofilm‐like aggregates that form in planta (Figure 3c). These data support the idea that plant‐produced SA is involved in reducing Pst aggregate size and numbers during infection.

### 2.6 Investigating the role of AlgU in suppressing plant‐produced intercellular SA

**FIGURE 4:**
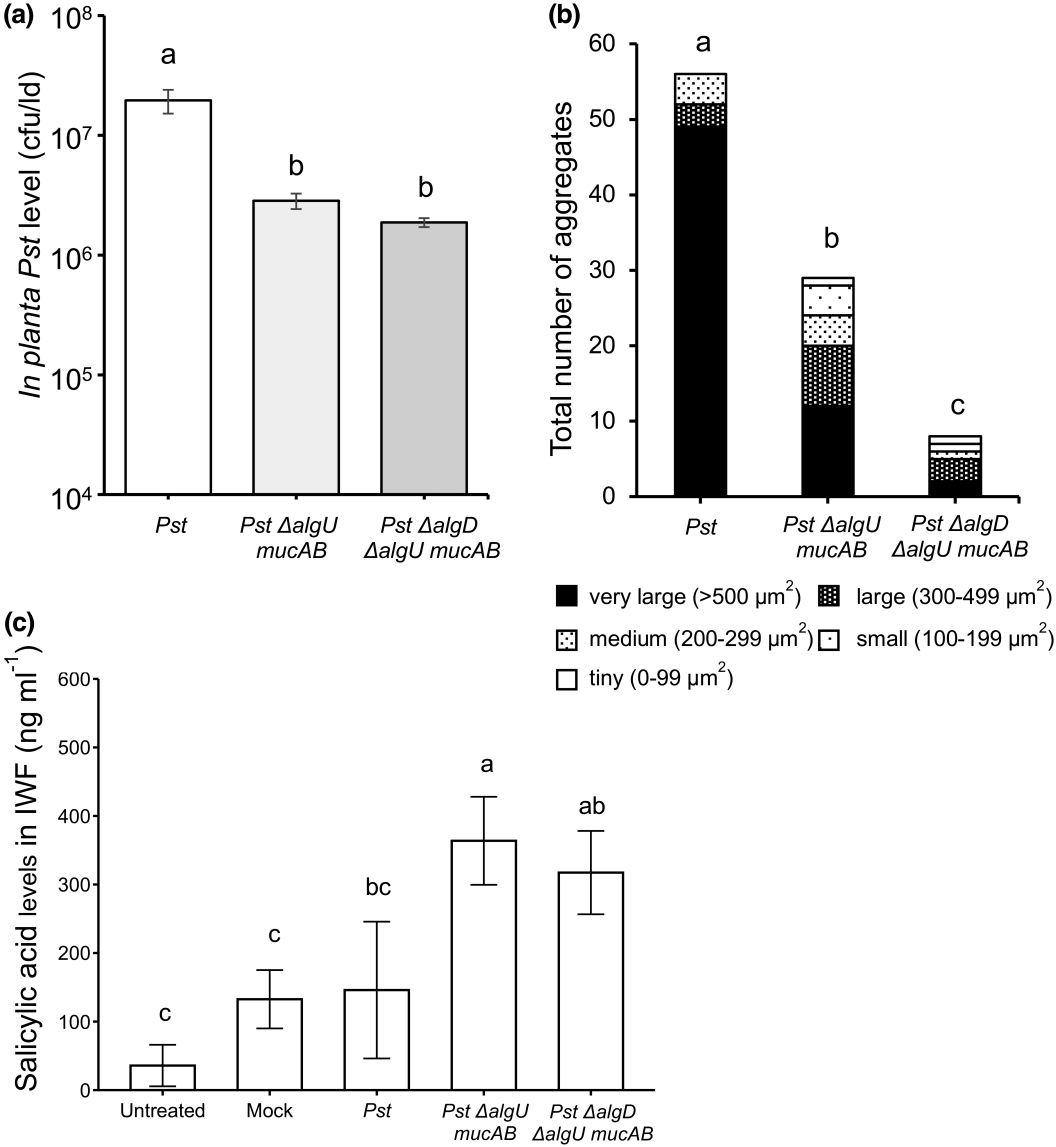
Multiplication and aggregate formation of *Pseudomonas syringae* pv. *tomato* (Pst), Pst Δ*algU* Δ*mucAB*, and Pst Δ*algD* Δ*algU* Δ*mucAB*. Leaves were inoculated with green fluorescent protein (GFP)‐expressing wild‐type virulent Pst or GFP‐expressing Pst mutants (Δ*algU* Δ*mucAB*, Δ*algD* Δ*algU* Δ*mucAB*) (10^6^ cfu/mL). (a) In planta bacterial quantification in Col‐0 at 48 h post‐inoculation (hpi). The *y* axis is cfu/leaf disc (ld) in log scale. Different letters indicate significant differences as determined using one‐way analysis of variance followed by Tukey's honestly significant difference test (*p* < 0.05). (b) At 48 hpi, aggregate formation was quantified by aggregate number and size in 40 fields of view for each strain and time point. Different letters indicate significant differences as determined using Kruskal–Wallis test. The experiments in panels (a) and (b) were performed one additional time with 10^6^ cfu/mL inoculum and once with 10^4^ cfu/mL inoculum (Figure S4) with similar results. (c) Salicylic acid (SA) accumulation was determined in intercellular washing fluids (IWF) collected at 24 hpi with Pst strains. Different letters indicate significant differences as determined using one‐way analysis of variance followed by Tukey's honestly significant difference test (*p* < 0.05). The experiment was performed one additional time with similar results (Figure S5).

The experiments above suggest that Pst aggregate formation is important for infection only in the absence of AlgU when Pst may be less able to inhibit SA‐mediated plant defence. To investigate this idea, IWFs were collected and SA levels were determined using an SA biosensor assay (DeFraia et al., 2008), along with determination of bacterial multiplication and aggregate formation in Col‐0 leaves of Pst Δ*algU* Δ*mucAB* triple mutants, which produce alginate but lack functional *AlgU*, and Pst Δ*algD* Δ*algU* Δ*mucAB* quadruple mutants, which lack both alginate biosynthesis and AlgU‐controlled T3SS effector gene expression. Similar results were obtained using a higher inoculum dose (Figure 4, 10^6^ cfu/mL) and a lower dose (Figure S4, 10^4^ cfu/mL). Both the triple and quadruple mutants multiplied to similar levels, approximately 10‐fold less than wild‐type Pst (Figure 4a). This was surprising given that Pst Δ*algD* Δ*algU* Δ*mucAB* formed very few aggregates (8), while Pst Δ*algU* Δ*mucAB* formed about three times more (29) and Pst formed 49 very large aggregates (56 in total) (Figure 4b). These data suggest that AlgU contributes to aggregate formation and in the absence of AlgU and AlgD, Pst formed very few aggregates. However, in response to all three Pst strains, Col‐0 leaves produced similarly low levels (c.120 to 350 ng/mL IWF) of intercellular SA compared to SA levels observed during the ARR response (1000–2000 ng/mL IWF) (Wilson et al., 2017), suggesting that like wild‐type Pst, both Pst Δ*algU* Δ*mucAB* and Pst Δ*algD* Δ*algU* Δ*mucAB* suppress SA‐mediated plant defence.

### 2.7 Examining Pst aggregate formation in PTI‐responding plants

**FIGURE 5:**
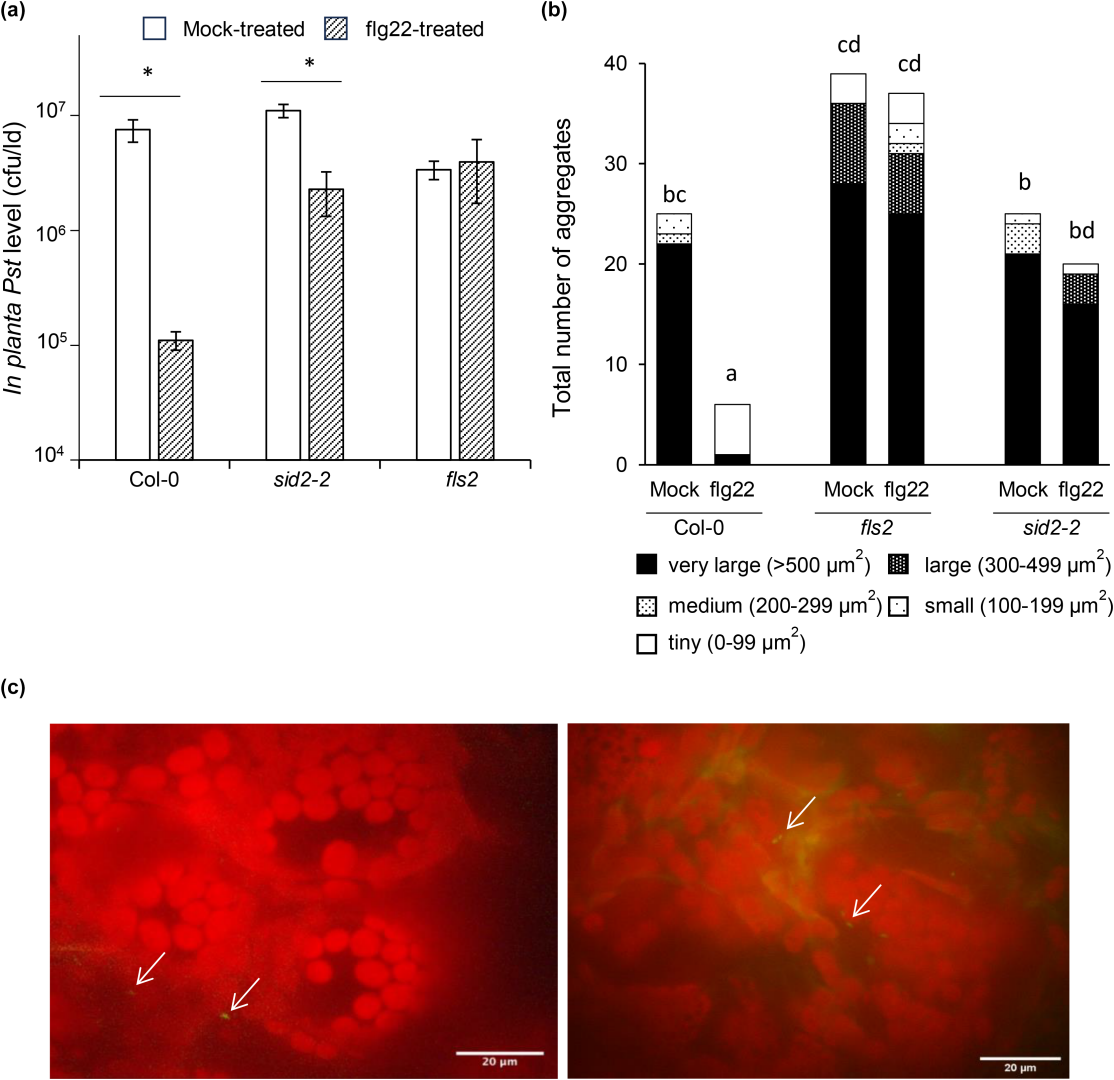
*Pseudomonas syringae* pv. *tomato* (Pst) aggregate formation in mock‐treated and pathogen‐associated molecular pattern‐triggered immunity (PTI)‐responding plants. Leaves were pressure‐infiltrated with 1 μM flg22 (flg22‐treated) or mock‐treated with water. Twenty‐four hours later, the same leaves were inoculated with virulent green fluorescent protein (GFP)‐expressing Pst. (a) In planta bacterial quantification of mock‐treated and flg22‐treated Col‐0, *fls2*, and *sid2‐2* at 72 h post‐inoculation (hpi). The *y* axis is cfu/leaf disc (ld) in log scale. Asterisks indicate significant differences (*p* < 0.05) as determined using Student's *t* test. (b) Aggregate formation was monitored by categorizing each microscopic field of view (40 fields of view per treatment) as with or without aggregates in mock‐treated and flg22‐treated Col‐0, *fls2*, and *sid2‐2* at 48 hpi. Different letters indicate significant differences in aggregate size distribution among groups as determined using the Kruskal–Wallis test (*p* < 0.05). This experiment (#5 in Table S2) was repeated seven additional times with similar results (Tables S2 and S3). (c) Visualization of GFP‐expressing Pst in PTI‐responding leaves. Leaves and bacteria were viewed at 1000× magnification under epifluorescence in plants treated with flg22 followed 1 day later by inoculation with 10^6^ cfu/mL Pst. Arrows mark examples of planktonic bacteria.

Given that Pst aggregate formation was reduced in mature ARR‐competent plants compared to young susceptible plants (Wilson et al., 2017), the effect of the PTI response on Pst aggregate formation was examined. PTI was initiated by 1 μM flg22 treatment of leaves, followed 1 day later by Pst inoculation (10^6^ cfu/ld) of the same leaves. A 67‐fold reduction in bacterial levels was observed in PTI‐responding compared to susceptible mock‐treated wild‐type Col‐0 plants, indicating that PTI was successfully established in this experiment (Figure 5a). In susceptible mock‐treated Col‐0, 22 very large aggregates were observed across 40 fields of view. In PTI‐responding (flg22‐treated) Col‐0 plants, five tiny aggregates and one very large aggregate were observed across 40 fields of view (Figure 5b). Similar results were observed in eight replicate experiments (Tables S2 and S3); however, the level of the PTI response as measured by the fold reduction between susceptible mock‐treated and PTI‐responding Col‐0 plants, and in terms of aggregate numbers and size, varied among experiments (Tables S2 and S3), indicating that the strength of the PTI response varied in experiments performed over two years (Table S4). These data indicate that successful infection by Pst (high bacterial levels) was associated with numerous Pst aggregates, whereas few aggregates and low Pst levels were observed in PTI‐responding leaves.

Pst biofilm‐like aggregate formation was also examined in mock‐ and flg22‐treated leaves of *fls2* plants (a flg22 receptor mutant that cannot mount flg22‐induced PTI) and of *sid2‐2* plants (which cannot accumulate SA) (Figure 5). A significant 1.5‐fold reduction in bacterial levels in flg22‐treated compared to mock‐treated *sid2‐2* was observed, indicating that a modest PTI response occurred in *sid2‐2* (Figure 5a), as expected as PTI is primarily SA‐dependent with some SA‐independent components (Mine et al., 2018). In terms of aggregate formation (Figure 5b), five aggregates (four tiny, one very large) were observed in PTI‐responding (flg22‐treated) Col‐0 plants compared to susceptible (mock‐treated) Col‐0 or *sid2‐2* leaves, both with 25 aggregates (21 and 22 very large, respectively). In *fls2* plants, which cannot initiate PTI in response to flg22 treatment, aggregate numbers were similar in both mock‐ (39) and flg22‐treated leaves (37). This experiment was repeated seven additional times, with similar results (Table S3), supporting the idea that suppression of bacterial biofilm‐like aggregate formation occurs during PTI.

### 2.8 Early intercellular SA accumulation is associated with reduced bacterial aggregate formation during PTI

Plant production of SA was associated with suppression of aggregate formation during Pst infection of *Arabidopsis* (Figures 3 and 5); therefore, it is possible that SA acts as an antimicrobial and antibiofilm agent in leaf intercellular spaces during PTI like it is thought to do during ARR (Wilson et al., 2017). Furthermore, Pst Δ*algD* Δ*algU* Δ*mucAB* appeared to be more sensitive to SA when cultured in leaf intercellular space‐mimicking medium, as the minimum inhibitory concentration (MIC) of growth was 1 mM SA in four of five experiments, while the MIC was 2 mM SA in two of three experiments for wild‐type Pst (Table S5). Moreover, the minimum bactericidal concentration was lower for Pst Δ*algD* Δ*algU* Δ*mucAB* (1 mM) than for wild‐type Pst (2 to 5 mM). These in vitro experiments suggest that SA plays a direct role in inhibiting bacterial multiplication and killing Pst, with a greater effect on Pst Δ*algD* Δ*algU* Δ*mucAB* than on wild‐type Pst.

**FIGURE 6:**
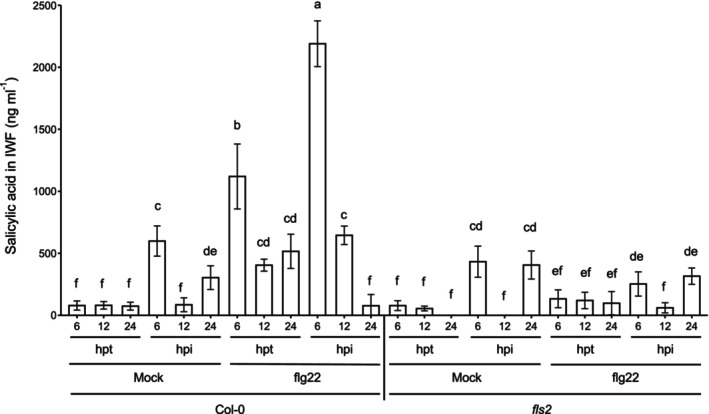
Salicylic acid (SA) accumulates in intercellular washing fluid (IWF) during pathogen‐associated molecular pattern‐triggered immunity. Young (3.5 weeks after germination) Col‐0 and *fls2* plants were mock‐ or 1 μM flg22‐treated, followed by *Pseudomonas syringae* pv. *tomato* inoculation (10^6^ cfu/mL) 24 h post‐treatment (hpt). In planta bacterial levels were measured at 72 h post‐inoculation (hpi). IWFs were collected at 6, 12, and 24 h post‐treatment (hpt) and hpi. IWF SA levels were quantified using an SA biosensor assay. Different letters indicate significant differences as determined using two‐way analysis of variance followed by Tukey's honestly significant difference test (*p* < 0.05). This experiment was performed five times in total with similar results.

Given that SA has antimicrobial effects on Pst growth (Table S5) and on biofilm formation by Pst in vitro (Wilson et al., 2017), intercellular SA may exert antimicrobial effects and inhibit biofilm formation during PTI. Therefore, intercellular SA accumulation was examined in PTI‐competent Col‐0 and *fls2* mutants in response to 1 μM flg22 treatment followed by Pst inoculation. We used the *fls2* mutant to confirm that PTI was initiated in response to flg22 treatment, and it also acted as the SA‐deficient control. Low bacterial levels were observed in flg22‐treated Col‐0 leaves, indicating a strong PTI response, while high bacterial levels were observed in the PTI‐defective mutant *fls2* with and without flg22 treatment, confirming that PTI was not induced in the *fls2* mutant (Figure S8). IWFs from mock‐treated Col‐0 and *fls2* leaves contained little intercellular SA (<50 ng/mL) at 6, 12, and 24 h post‐treatment (hpt) (Figure 6). In addition, low levels of SA (<550 ng/mL) were detected in IWFs collected at 6 and 24 h post‐inoculation (hpi) from mock‐treated Col‐0 and *fls2* leaves (Figure 6). Little SA was detected at 12 hpi with Pst in IWFs collected from mock‐treated Col‐0 and *fls2* leaves (Figure 6). This is consistent with previous work in which young, susceptible Col‐0 accumulated little intercellular SA in response to Pst (Carviel et al., 2014; Wilson et al., 2017). Moreover, mock‐treated plants (Col‐0 and *fls2*) that accumulated little SA in leaf intercellular spaces (Figure 6) were susceptible to Pst, as indicated by high in planta bacterial levels (Figure S8). Unlike mock‐treated plants, IWFs from flg22‐treated PTI‐responding Col‐0 leaves contained high levels of SA at 6 hpt (1000 ng/mL) and 6 hpi (2050 ng/mL), demonstrating that SA accumulated in leaf intercellular spaces in flg22‐treated Col‐0 leaves (Figure 6). In addition, IWFs from flg22‐treated Col‐0 plants contained SA at 12 hpi (650 ng/mL), whereas little SA was detected in IWFs (<50 ng/mL) in other treatment groups. In contrast, IWFs from flg22‐treated PTI‐defective *fls2* accumulated little SA (<50 ng/mL), like mock‐treated controls, indicating that intercellular SA accumulation is a component of PTI because PTI was initiated by flg22 treatment in Col‐0, but not *fls2* (Figure 6, Figure S8). Pst was observed to form few biofilm‐like aggregates in PTI‐responding Col‐0 leaves in numerous experiments (Figure 5b, Tables S2 and S3), and combined with our SA analysis (Figure 6), this demonstrates that early SA accumulation during PTI is associated with a reduction in Pst biofilm‐like aggregate formation.

## 3 DISCUSSION

### 3.1 Biofilm‐like aggregate formation is associated with Pst success in planta

Pst colonies and aggregates have been observed in tomato and *Arabidopsis* (Badel et al., 2002; Boureau et al., 2002; Varvaro et al., 1993; Whalen et al., 1991), leading to the idea that Pst forms biofilms as part of its infection strategy. However, these studies provided no quantitative data to indicate an association between Pst aggregate formation and proliferation in planta. In the present study, virulent Pst multiplied to high levels and formed many large biofilm‐like aggregates in leaf intercellular spaces of susceptible *Arabidopsis* leaves. Moreover, Pst biofilm‐like aggregate formation was positively correlated with Pst levels in planta, suggesting that biofilm‐like aggregate formation contributes to Pst success in planta.

The loss of alginate biosynthesis did not affect Pst Δ*algD* multiplication in *Arabidopsis* leaves compared to wild‐type Pst, consistent with experiments in which Pst Δ*algD* multiplied like wild‐type Pst in tomato and 2‐week‐old *Arabidopsis* seedlings (Ishiga et al., 2018), suggesting that the ability to produce alginate is not important for infection of susceptible *Arabidopsis* by virulent Pst. We reasoned that Pst Δ*algD* was able to colonize *Arabidopsis* despite a reduced ability to form aggregates (this work) because it was still able to suppress plant defence with its intact T3SS (Markel et al., 2016). To address this, we investigated the Pst Δ*algD* Δ*algU* Δ*mucAB* mutant with reduced virulence due to loss of AlgU, a sigma factor that regulates alginate biosynthesis genes including *AlgD* and the T3SS gene *HrpL*, which controls expression of some T3SS effector genes (*HopY1*, *AvrPtoB*, *AvrPto*, *HopE1*) (Ishiga et al., 2018; Markel et al., 2016). Pst Δ*algD* Δ*algU* Δ*mucAB* displayed reduced bacterial multiplication in *Arabidopsis* leaves compared to wild‐type Pst (this work; Markel et al., 2016) and formed fewer and smaller aggregates compared to wild‐type Pst, suggesting that the *Arabidopsis* defence response was effective in suppressing Pst Δ*algD* Δ*algU* Δ*mucAB* biofilm‐like aggregate formation. The AlgU contribution to suppression of intercellular SA accumulation during plant defence in response to Pst infection was examined by comparing Pst Δ*algD* Δ*algU* Δ*mucAB* to Pst Δ*algU* Δ*mucAB*. Both mutants accumulated little SA, just like wild‐type Pst, suggesting that AlgU does not regulate the expression of Pst genes that contribute to suppression of SA‐mediated plant defence.

Given that plant‐produced SA accumulated to similarly low levels in response to wild‐type Pst and both *algU* mutants, AlgU's other proposed functions may contribute to Pst aggregate formation and multiplication. For example, it has been observed that AlgU down‐regulates flagellin gene expression, which may allow Pst to avoid flagellin‐mediated PTI detection (Bao et al., 2020) and may also promote the switch from planktonic to sessile bacterial cells to initiate biofilm formation during infection (Bao et al., 2020; Markel et al., 2016, 2018; Wang et al., 2021). These ideas are consistent with the results presented in this work, in which Pst Δ*algD* Δ*algU* Δ*mucAB* and Pst Δ*algU* Δ*mucAB* formed fewer and smaller aggregates compared to wild‐type Pst in *Arabidopsis* leaves, suggesting that AlgU is an important regulator of biofilm formation by promoting alginate production and down‐regulating flagellin gene expression during Pst infection of *Arabidopsis* (Wang et al., 2021).

### 3.2 Biofilm‐like aggregates are surrounded by biofilm components

Although biofilm‐like aggregates were observed in planta (Bogino et al., 2013) and extracellular material surrounded Pst aggregates cultured in vitro (Farias & Olmedilla, 2019) and at infection sites in *Arabidopsis* (Whalen et al., 1991), the composition of this extracellular material was not determined in planta. We demonstrated that Pst‐GFP aggregates colocalized with DAPI, ConA‐TRITC, and calcofluor white signals, suggesting that Pst aggregates were embedded in a matrix of extracellular eDNA and polysaccharides to form biofilms in the leaf intercellular space during infection of *Arabidopsis*. Additional experiments are required to confirm that these components (eDNA, carbohydrates) are produced by Pst bacterial cells.

### 3.3 PTI is associated with SA‐dependent reduction in biofilm‐like aggregates

Wild‐type Pst formed many large aggregates in susceptible plants, including *fls2* mutants unable to initiate flg22‐triggered PTI, while few aggregates were observed in PTI‐responding plants, suggesting that flg22‐induced PTI suppresses Pst biofilm‐like aggregate formation. This idea was supported by the positive correlation between in planta bacterial levels and biofilm‐like aggregate formation across many experiments, such that PTI‐responding plants supported low Pst levels and few Pst biofilm‐like aggregates. Moreover, transcriptome studies revealed that bacterial survival and virulence genes, including genes for sulphur import, iron scavenging, and alginate biosynthesis, were down‐regulated during PTI (Lovelace et al., 2018; Nobori et al., 2018). Collectively, these studies (this work, Lovelace et al., 2018; Nobori et al., 2018) suggest that the PTI response affects numerous bacterial processes.

Given that Wilson et al. (2017) provided evidence supporting the idea that SA acts as an antimicrobial and antibiofilm agent during ARR, we predicted that (1) SA would perform a similar role during PTI, and (2) Pst Δ*algD* Δ*algU* Δ*mucAB* with a reduced ability to suppress plant defence would be less successful in wild‐type plants with intact SA‐mediated defence. As expected, in SA‐deficient *sid2‐2*, Pst Δ*algD* Δ*algU* Δ*mucAB* bacterial levels increased approximately 100‐fold and many large aggregates formed in comparison to wild‐type Col‐0 leaves, suggesting that plant‐produced SA negatively affects Pst Δ*algD* Δ*algU* Δ*mucAB* multiplication and aggregate formation in wild‐type leaves. These data suggest that plant‐produced SA is involved in reducing Pst aggregate size and aggregate numbers during infection by Pst. This is consistent with the observation that smaller *P. aeruginosa* biofilm‐like aggregates were detected on SA‐hyperaccumulating *Arabidopsis* mutant roots (*lox2* and *cpr5‐2*) compared to wild‐type roots (Prithiviraj et al., 2005). The data presented in this work and by Prithiviraj et al. (2005) suggest that the ability to produce SA is important for defence against biofilm‐forming bacteria.

### 3.4 SA accumulates in leaf intercellular spaces and is associated with reduced Pst multiplication and biofilm‐like aggregate formation during PTI

PTI‐responding Col‐0 leaves accumulated high levels of SA in leaf intercellular spaces and few Pst aggregates were observed. In contrast, susceptible plants (mock‐treated Col‐0 and mock‐ or flg22‐treated *fls2*) inoculated with wild‐type Pst accumulated little intercellular SA and Pst formed many large aggregates in leaf intercellular spaces. Moreover, accumulation of SA in leaf intercellular spaces was associated with reduced Pst multiplication and reduced biofilm‐like aggregate formation during PTI compared to a susceptible interaction, suggesting that intercellular SA accumulation negatively impacts bacterial success in planta. Although SA suppressed Pst in wild‐type plants (Bauters et al., 2021), Pst multiplication and aggregate formation were greater in SA‐deficient *sid2‐2* compared to wild‐type Col‐0 (this work), indicating that Pst benefited from growing in SA‐deficient plants. This makes sense given that intercellular SA still accumulated, although to reduced levels, in mock‐treated susceptible compared to flg22‐induced PTI‐responding plants (this work).

A role for intercellular SA accumulation was first observed in mature plants during ARR in response to Pst (Cameron & Zaton, 2004; Carviel et al., 2009; Wilson et al., 2017). Reduction of SA levels in leaf intercellular spaces by infiltration of salicylate hydroxylase resulted in a reduced ARR response and infiltration of SA into intercellular spaces rescued the ARR response in ARR‐defective mutants (Cameron & Zaton, 2004). Biofilm‐like aggregate formation was reduced in mature ARR‐competent plants, but not in mature ARR‐incompetent mutant plants, suggesting that suppression of biofilm‐like aggregate formation is an important component of ARR (Wilson et al., 2017). The peak concentration of intercellular SA (c.2000 ng/mL) in PTI‐responding leaves was similar to the intercellular SA levels in ARR‐competent leaves (Wilson et al., 2017). Pst multiplication and aggregate formation were reduced in PTI‐responding leaves that accumulated intercellular SA, suggesting that SA plays an antimicrobial and antibiofilm role in leaf intercellular spaces during PTI. Furthermore, high levels of intercellular SA accumulated in ETI‐responding plants, suggesting that intercellular SA accumulation also contributes to ETI (Carviel et al., 2014). These studies (this work, Carviel et al., 2014; Wilson et al., 2017) support the idea that SA acts as an antimicrobial and antibiofilm agent in leaf intercellular spaces during PTI, ETI, and ARR.

During *Agrobacterium* infections, plant‐produced SA is thought to act as a quorum‐quenching agent as demonstrated by reduced *Agrobacterium* quorum‐sensing signals and reduced virulence gene expression in SA‐competent versus SA‐deficient plants (Subramoni et al., 2014). SA is also thought to act as a modulator of virulence in human‐pathogenic *P. aeruginosa* (Bandara et al., 2006) and SA concentrations of 1.5 mM suppress quorum sensing‐related gene expression in *P. aeruginosa* grown in culture (Yang et al., 2009). SA concentrations greater than 1.8 mM also suppress biofilm formation by *Escherichia coli* grown in culture (Cattò et al., 2017). Moreover, SA was identified in a screen for AHL synthase inhibitors (Chang et al., 2014), suggesting that SA can directly target quorum‐sensing signal production and downstream pathogenicity and biofilm responses in *E. coli* and *P. aeruginosa*. It is tempting to speculate that intercellular SA accumulation during ARR, ETI, and PTI may interfere with Pst quorum sensing, resulting in inhibition of biofilm formation. Additional studies are required to understand if and how intercellular SA accumulation contributes to the suppression of bacterial biofilm‐like aggregate formation. Given current limitations in observing all fields of view in the large three‐dimensional space of a leaf disc combined with the fact that there are very few bacterial cells or aggregates at early times in a PTI‐responding leaf, it is not possible to determine if intercellular SA produced during PTI acts first to negatively impact Pst multiplication, acts first to negatively impact Pst aggregate formation, or acts simultaneously on both.

Our work demonstrates that biofilm‐like aggregate formation is associated with successful Pst infection of *Arabidopsis*. Common biofilm matrix components colocalized with Pst biofilm‐like aggregates in leaf intercellular spaces, supporting the idea that Pst cells form biofilms during infection of *Arabidopsis*. Intercellular SA accumulated during the PTI response that was associated with the suppression of Pst biofilm‐like aggregate formation, suggesting that suppression of biofilm formation is an important component of PTI. Not only does plant‐produced intracellular SA act as a signal to up‐regulate plant defence genes (Mine et al., 2018), but SA also accumulates in leaf intercellular spaces, where it may act as an antimicrobial and antibiofilm agent during multiple plant defences: PTI (this work), ARR (Wilson et al., 2017), and ETI (Carviel et al., 2014).

## 4 EXPERIMENTAL PROCEDURES

### 4.1 Imaging of Pst in intercellular spaces by fluorescence microscopy

Leaves were infiltrated with 1 mM flg22 peptide (PhytoTech Labs #P6622) or mock‐treated with sterile water. Twenty‐four hours later, the same leaves were inoculated with 10^6^ cfu/mL Pst pDSK‐GFPuv (Wang et al., 2007). After 24, 48, and 72 h, leaves were cut at the petiole and sections of the lower epidermis were removed using tape and mounted in water on a glass slide with the epidermis‐less surface facing upwards. Slides were imaged immediately using an Eclipse E800 microscope (Nikon) fitted with a DS‐Fi1 camera head (Nikon) and the DS‐U3 control unit using 100× oil immersion lenses and a B‐2A filter cube. To score cell types (planktonic or aggregated) and aggregate sizes, tissue preparation and imaging were performed by different individuals so that scoring was blind. Aggregate size was measured using ImageJ software (US National Institutes of Health).

At 24 hpi, few planktonic bacterial cells or aggregates were observed in leaf intercellular spaces as bacterial levels were low at this time point (c.10^4–5^ cfu/ld) (Figure S7). Additionally, the leaf area observed in each field of view at 1000× magnification was very small (1.2 × 10^−8^ m^2^ or 1.2 × 10^4^ μm^2^ per field of view). At 48 hpi, bacterial levels increased 100‐fold, to approximately 10^6–7^ cfu/ld, making it possible to observe aggregates and planktonic cells in many fields of view (Figure S7). At 72 hpi, in planta bacterial levels reached approximately 10^7^ cfu/ld and large biofilm‐like aggregates surrounded by many planktonic bacteria were often observed over the entire field of view in susceptible interactions, including in mock‐treated Col‐0 plants inoculated with Pst and mock‐ or flg22‐treated *fls2* and *sid2‐2* plants (Figure S7). Moreover, at 72 hpi, some plant cell death was observed as some Pst cells switched to the necrotrophic phase (Xin & He, 2013). Based on these observations, all experiments were conducted at 48 hpi.

### 4.2 Extracellular matrix staining and colocalization analysis

Plants were inoculated with 10^6^ cfu/mL Pst pDSK‐GFPuv (Wang et al., 2007). At 48 h, leaves were cut at the petiole and sections of the lower epidermis were removed using tape and mounted on a glass slide with the epidermis‐less surface facing upwards. Different leaves were stained to detect eDNA or extracellular polysaccharides. A UV filter (435–485 nm) was used during florescence microscopy with an Axioscope epifluorescence microscope (Zeiss) fitted with an AxioCam ICm 1 monochrome camera (Zeiss) and a C‐Mount lens. See File S1 for more details.

### 4.3 IWF collection

The IWF collection method was adapted from Baker et al. (2012) and O'Leary et al. (2014)—see this reference for a video demonstration of the technique. Three pools of 8–12 leaves were cut at the petiole, weighed, and then vacuum‐infiltrated with water inside a 60‐mL syringe until they appeared completely water‐soaked. Leaves were blotted dry, stacked between Parafilm sheets, rolled around a 1‐mL pipette tip, secured with a twist‐tie, and placed inside the bottom third of a 60‐mL syringe fitted to a 1.5‐mL centrifuge tube with the petioles facing up. Leaves were centrifuged using a swinging bucket rotor at 600 *g* for 15 min at room temperature to collect the IWFs. The IWFs were centrifuged for an additional 5 min at 13,000 *g*, transferred to fresh tubes, weighed, and stored at −80°C. The remaining pellets (chlorophyll sometimes visible) were resuspended in 1 mL ethanol and measured with a spectrophotometer at 664 and 700 nm to assess contamination of IWFs with cellular contents (Baker et al., 2012). In a representative experiment, the chlorophyll levels in IWFs were 0.85% of the corresponding leaf tissue, indicating that the IWFs had minimal cellular contamination.

### 4.4 SA quantification in IWFs

IWFs from leaves were collected at 12, 24, and 48 hpi (see above). ADPWH_*lux* is a nonpathogenic soil bacterium modified to produce luciferase proportional to the amount of SA present (Huang et al., 2006) and was used to measure free SA (DeFraia et al., 2008). The method is briefly described as follows. ADPWH_*lux* was grown overnight in Luria–Bertani (LB) medium with shaking at 37°C and then diluted to OD_600_ = 0.4 with fresh LB, followed by incubation with IWF samples and SA standard curve samples for 1 h at 37°C in an opaque 96‐well plate (Corning no. 3915). Luminescence was measured on a BioTek plate reader at 490 nm. An SA standard curve was plotted and luminescence data were converted to SA concentrations using the SA standard curve.

See File S1 for additional methods and Table S1 for bacterial strains.

## CONFLICT OF INTEREST STATEMENT

All authors declare no conflicts of interest.

## Supporting information


**Figure S1.** Colocalization analysis of GFP‐expressing *Pseudomonas syringae* pv. *tomato* (Pst) and ConA‐TRITC and calcofluor white (CFW) stains.

**Figure S2.** Quantification of aggregate size and number in *Pseudomonas syringae* pv. *tomato* (Pst) and Pst Δ*algD* in wild‐type Col‐0 and *sid2‐2*.

**Figure S3.** Growth of alginate‐deficient mutants *Pseudomonas syringae* pv. *tomato* (Pst) ∆*algD* and Pst ∆*algD* ∆*algU* ∆*mucAB* in *hrp*‐inducing minimal (HIM) medium.

**Figure S4.** Multiplication and aggregate formation of *Pseudomonas syringae* pv. *tomato* (Pst), Pst Δ*algU* Δ*mucAB*, and Pst Δ*algD* Δ*algU* Δ*mucAB* inoculated at a low dose.

**Figure S5.** Salicylic acid levels in intercellular washing fluids (IWFs) of *Pseudomonas syringae* pv. *tomato* (Pst)‐, Pst Δ*algU* Δ*mucAB*‐, and Pst Δ*algU* Δ*mucAB* Δ*algD*‐inoculated leaves.

**Figure S6.** Quadratic correlation between bacterial levels and aggregation.

**Figure S7.** Bacterial levels and biofilm‐like aggregate formation during *Pseudomonas syringae* pv. *tomato* infections in planta.

**Figure S8.** Bacterial multiplication during flg22‐induced PAMP‐triggered immunity.

**Method S1.** *Arabidopsis* plant lines and growth conditions.
**METHOD S2** Bacterial strains and transformation.
**METHOD S3** PAMP‐triggered immunity assays and in planta quantification of bacterial levels.
**METHOD S4** Extracellular matrix staining and colocalization analysis.
**METHOD S5** Statistical analysis.
**METHOD S6** In vitro antibacterial growth curve assays.
**TABLE S1** Bacterial strains.

**Table S2.** The PAMP‐triggered immunity response varies across experiments.

**Table S3.** Percentage of fields of view (FOVs) with *Pseudomonas syringae* pv. *tomato* aggregates in flg22‐ and mock‐treated leaves.

**Table S4.** PAMP‐triggered immunity responses across seasons.

**Table S5.** Effect of salicylic acid on *Pseudomonas syringae* pv. *tomato* (Pst) GFP and Pst mutants cultured in apoplast‐mimicking medium (HIM**).

## Data Availability

The data that support the findings of this study are available from the corresponding author upon request.
