# Supplementary material for: Exploring Pseudomonas syringae pv. tomato biofilm‐like aggregate formation in susceptible and PTI‐responding Arabidopsis thaliana
Source: Mol Plant Pathol. 2023 Nov 21;25(1):e13403. doi: 10.1111/mpp.13403 (PMC10799205; doi:10.1111/mpp.13403)
Supplement: Supplementary file 1 — Figure S1. Colocalization analysis of GFP‐expressing Pseudomonas syringae pv. tomato (Pst) and ConA‐TRITC and calcofluor white (CFW) stains. [file MPP-25-e13403-s012.pdf]

Supporting Information for Exploring *Pseudomonas syringae* pv *tomato* aggregate formation in susceptible and PTI-responding *Arabidopsis thaliana*

Supplementary Figures

Fig. S1. Co-localisation analysis of GFP-expressing *Pst* and ConA-TRITC and Calcofluor white (CFW) stains. The epidermis was peeled from leaves inoculated with 10^6 cfu/ml *Pst*-GFP at 48 hpi, then stained with ConA-TRITC to visualize α-polysaccharides (Cy5 filter, 663-738 nm) and calcofluor white (CFW) to visualize β-polysaccharides (UV filter, 435-485 nm) using a fluorescence microscope (representative images shown in Fig 2B). For each image of an aggregate, 2% of pixels were set to black and 0.01% set to fully saturated and images were merged. Co-localization was calculated by comparing the pixel-matched intensity values of GFP to the pixel intensity values of either ConA-TRITC or CFW using Pearson's correlation coefficient (a), Li's intensity correlation quotient (b), or Mander's split co-occurrence (c-d). Lower, middle, and upper horizontal lines in boxplots represent the 1st, 2nd and 3rd quartiles (n=9). Different letters indicate significant differences using a one-way ANOVA (Tukey's HSD, $p<0.05$). Experiment was completed twice with similar results. e-f) Fluorescence images of uninoculated leaf with epidermis peeled and stained with calcofluor white (e) and ConA-TRITC (f).

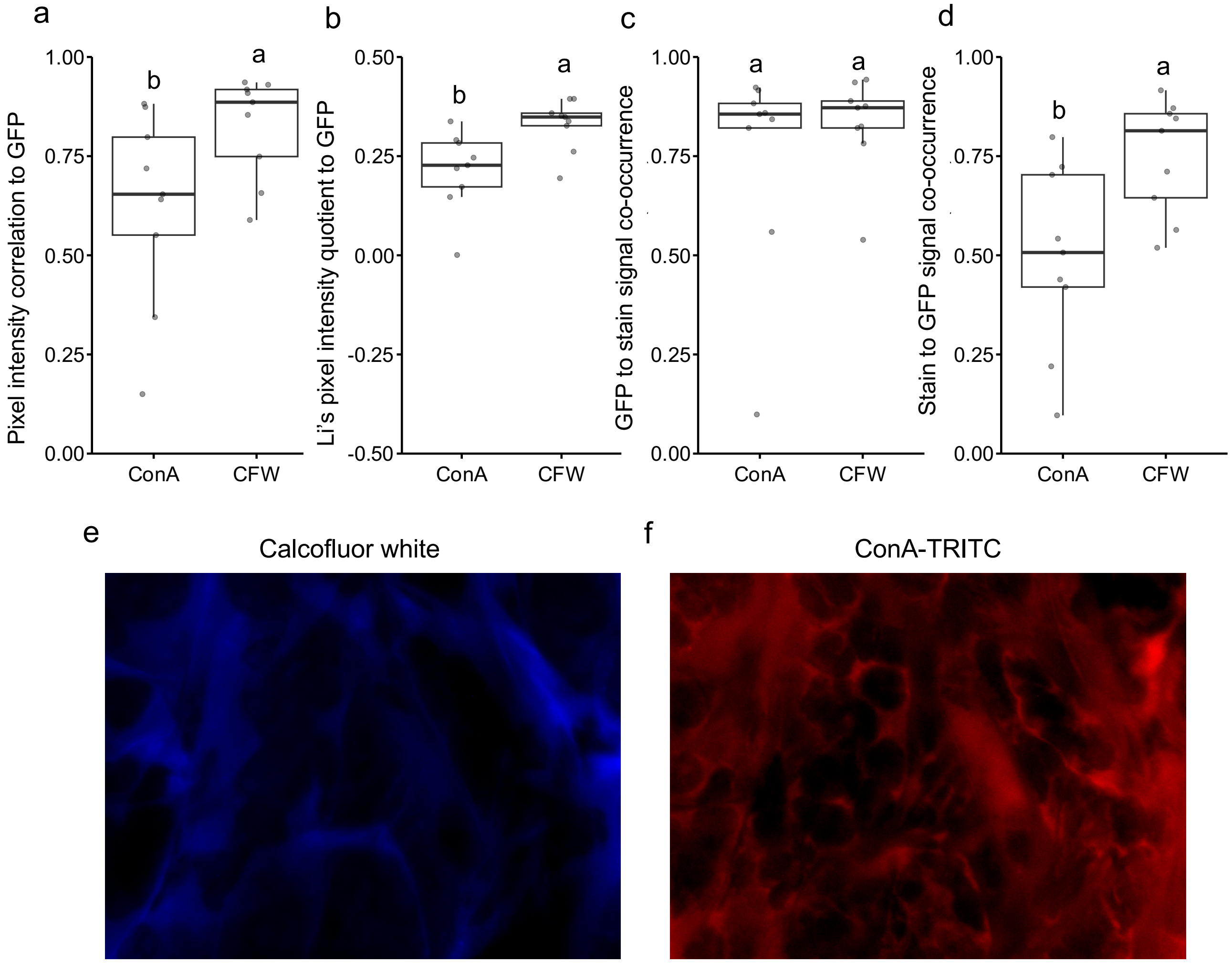

Detailed Discussion of Figure S1 (summarized in Results section)

Nine aggregates were stained, imaged and analyzed using all three methods.Pearson's correlation coefficient and Li's intensity quotient analysis showed a positive correlation between ConA-TRITC and calcofluor white signals with GFP (Fig. **S1ab**), suggesting ConA-TRITC and calcofluor white signals localized to *Pst*-GFP aggregates. Pearson's correlation coefficient and Li's intensity quotient analysis displayed lower ConA-TRITC with GFP co-localization compared to calcofluor white with GFP co-localization (Fig. **S1ab**) suggesting calcofluor white more readily stains *Pst* aggregates than ConA-TRITC. Mander's split co-occurance analysis showed a large proportion of pixels above the threshold for the GFP channel were also above the threshold for both the ConA-TRITC and calcofluor white channels (Fig. **S1c**), demonstrating that both ConA-TRITC and calcofluor white co-localize to *Pst*-GFP. If ConA-TRITC and calcofluor white preferentially bind *Pst*-GFP aggregates over plant cell walls we would expect a majority of pixels above the threshold for the ConA-TRITC and calcofluor white channels would also be above the threshold for the GFP channel. Therefore we examined the Mander's split co-occurrence of either ConA-TRITC and calcofluor white to GFP (Fig. **S1d**), and there was a positive proportion of pixels above the threshold for ConA-TRITC and calcofluor white that overlapped with GFP pixels above the threshold (Fig. **S1d**). The positive proportion of overlapping pixels suggests ConA-TRITC and calcofluor white preferentially stain *Pst* aggregates. A lower proportion of ConA-TRITC and calcofluor white pixels overlapped with the GFP than GFP overlapped with either of the stains, this was expected as calcofluor white is known to stain cellulose in plant cell walls (Maeda & Ishida, 1967; Bidhendi *et al.*, 2020) and was observed to stain cell wall components in images of uninoculated leaves (Fig. **S1e**). Similarly ConA-TRITC was observed to stain mesophyll cell walls (Fig. **S1f**). Overall, these analyses demonstrate that ConA-TRITC and calcofluor white colocalized with GFP-expressing *Pst* aggregates, suggesting aggregates of *Pst* are surrounded by an extracellular matrix of α- and β-polysaccharides.
