# Supplementary material for: Exploring Pseudomonas syringae pv. tomato biofilm‐like aggregate formation in susceptible and PTI‐responding Arabidopsis thaliana
Source: Mol Plant Pathol. 2023 Nov 21;25(1):e13403. doi: 10.1111/mpp.13403 (PMC10799205; doi:10.1111/mpp.13403)

Figure S2. Quantification of aggregate size and number in *Pst & Pst ΔalgD* in wild-type Col-0 & *sid2-2*. Col-0 and *sid2-2* leaves were inoculated with GFP-expressing wild-type virulent *Pst* or GFP-expressing alginate biosynthesis mutant *Pst ΔalgD.* a) *In planta* bacterial quantitation of wild-type *Pst*-inoculated and *Pst algD*-inoculated Col-0 at 48 hpi, y axis is in log scale. Different letters indicate significant differences using a two-way ANOVA (Tukey's HSD, $p < 0.05$). b) ImageJ was used to calculate the area of each aggregate present in each of 40 fields of view at 48 hpi. The percent of aggregates in each size category was calculated relative to the total number of aggregates. The total number of aggregates in 40 fields of view is indicated above each column. c) example of large, medium, small and tiny aggregates of *Pst ΔalgD* in leaf intercellular spaces at 48hpi. This experiment (a &b) was repeated 2 times with similar results.

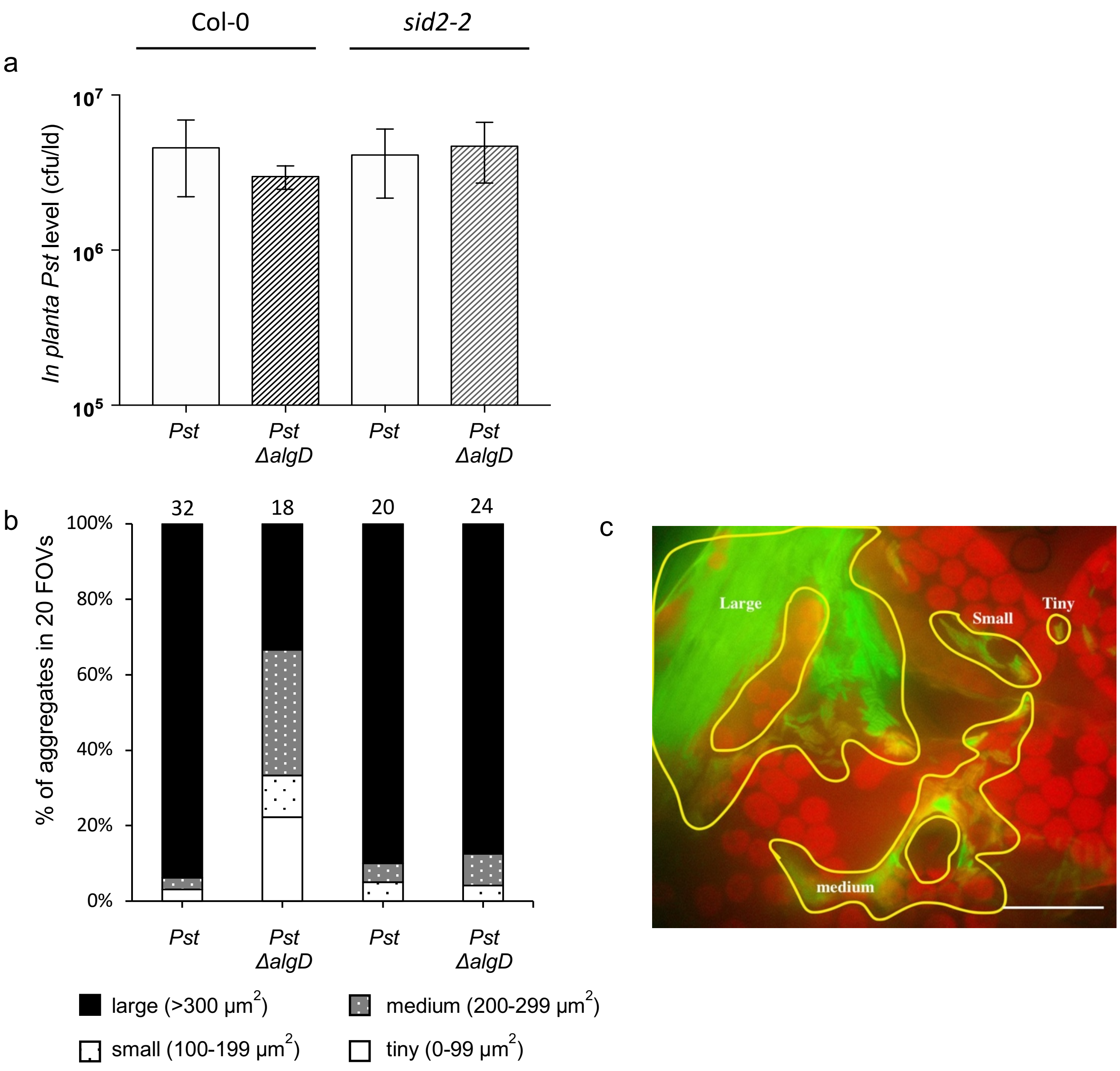

Supplement: Supplementary file 2 — Figure S2. Quantification of aggregate size and number in Pseudomonas syringae pv. tomato (Pst) and Pst ΔalgD in wild‐type Col‐0 and sid2‐2. [file MPP-25-e13403-s007.pdf]
