# Supplementary material for: Exploring Pseudomonas syringae pv. tomato biofilm‐like aggregate formation in susceptible and PTI‐responding Arabidopsis thaliana
Source: Mol Plant Pathol. 2023 Nov 21;25(1):e13403. doi: 10.1111/mpp.13403 (PMC10799205; doi:10.1111/mpp.13403)

Fig. S3. Growth of alginate deficient mutants *Pst ΔalgD* and *Pst ΔalgD ΔalgU ΔmucAB* in *hrp*-inducing minimal (HIM) media. *Pst* strains was grown at 26 °C in HIM with shaking for 72 hours in a 96-well plate. Each data point represents the mean of 4 wells and error bars indicate standard deviation. Asterisks indicate statistically significant differences compared to wild type *Pst* (p-value <0.05, Student's T-test). Experiments were performed twice with similar results.

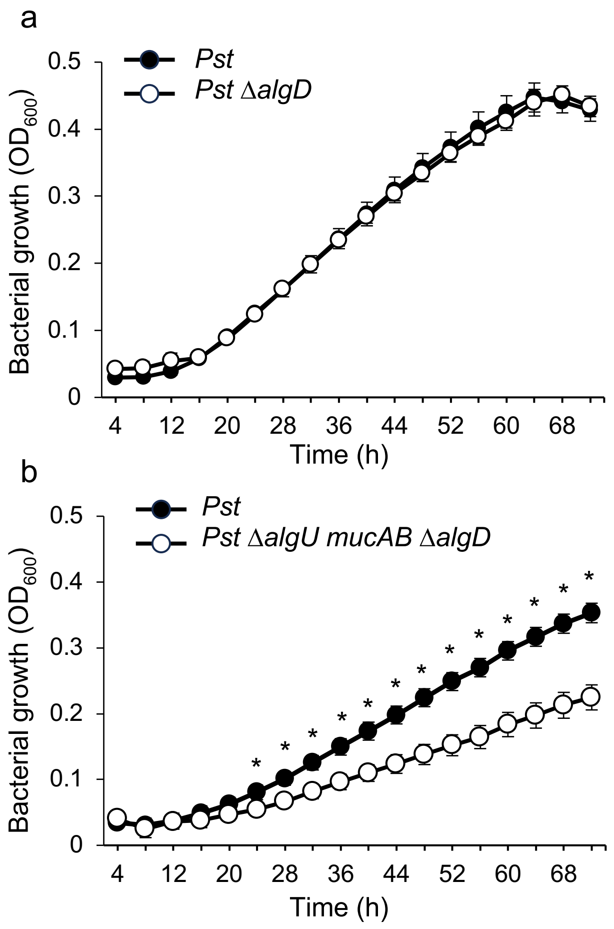

Supplement: Supplementary file 3 — Figure S3. Growth of alginate‐deficient mutants Pseudomonas syringae pv. tomato (Pst) ∆algD and Pst ∆algD ∆algU ∆mucAB in hrp‐inducing minimal (HIM) medium. [file MPP-25-e13403-s006.pdf]
