# Supplementary material for: Exploring Pseudomonas syringae pv. tomato biofilm‐like aggregate formation in susceptible and PTI‐responding Arabidopsis thaliana
Source: Mol Plant Pathol. 2023 Nov 21;25(1):e13403. doi: 10.1111/mpp.13403 (PMC10799205; doi:10.1111/mpp.13403)

**Fig. S4 Multiplication and aggregate formation of *Pst, Pst ΔalgUΔmucAB & Pst ΔalgDΔalgUΔmucAB* inoculated at a low dose.** Col-0 leaves were inoculated with a low dose ($10^4$ cuf/ml) of GFP-expressing wild-type *Pst* or GFP-expressing *Pst* mutants ***(ΔalgUΔmucAB, ΔalgDΔalgUΔmucAB).*** a) *In planta* bacterial quantitation in Col-0 at 1,2,3,4,5,6,7 dpi, y axis is in log scale. Different letters indicate significant differences using Two-way ANOVA (Tukey's HSD, p<0.05). b) Aggregate formation was monitored by categorizing each microscopic field of view (40 FOV per treatment) as containing no bacteria, only planktonic bacteria, only bacterial aggregates, or both planktonic and bacterial aggregates at 1 to 7 dpi. No aggregates were observed at 1 to 4 dpi or at 6 dpi.Different letters indicate significant differences using Kruskal-Wallis test. This experiment consisting of panels a and b was repeated 2 times with similar results.

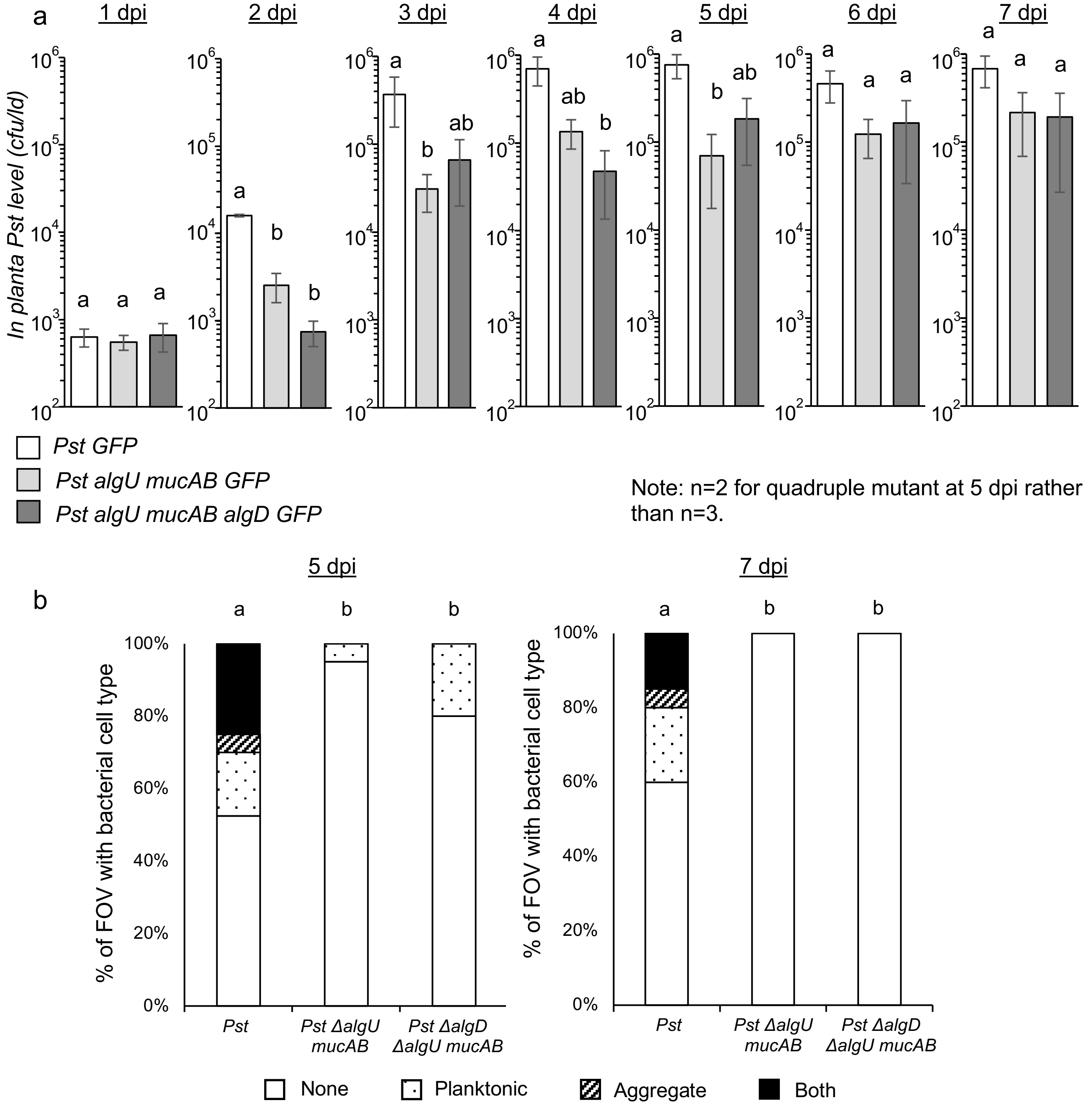

Supplement: Supplementary file 4 — Figure S4. Multiplication and aggregate formation of Pseudomonas syringae pv. tomato (Pst), Pst ΔalgU ΔmucAB, and Pst ΔalgD ΔalgU ΔmucAB inoculated at a low dose. [file MPP-25-e13403-s001.pdf]
