# Supplementary material for: Exploring Pseudomonas syringae pv. tomato biofilm‐like aggregate formation in susceptible and PTI‐responding Arabidopsis thaliana
Source: Mol Plant Pathol. 2023 Nov 21;25(1):e13403. doi: 10.1111/mpp.13403 (PMC10799205; doi:10.1111/mpp.13403)

Fig. S5. Salicylic acid levels in intercellular washing fluids (IWFs) of *Pst, Pst ΔalgU mucAB & Pst ΔalgU mucAB ΔalgD* inoculated leaves. Leaves were inoculated with 10^6 cfu/ml GFP-expressing wild-type virulent *Pst* or GFP-expressing *Pst* mutants (*ΔalgU mucAB , ΔalgU mucA ΔalgD*). SA accumulation was determined in IWFs collected at 6, 12, and 24 hpi with indicated *Pst* strain. Different letters indicate significant differences using a one-way ANOVA (Tukey's HSD, p<0.05). Experiment was repeated once with similar results.

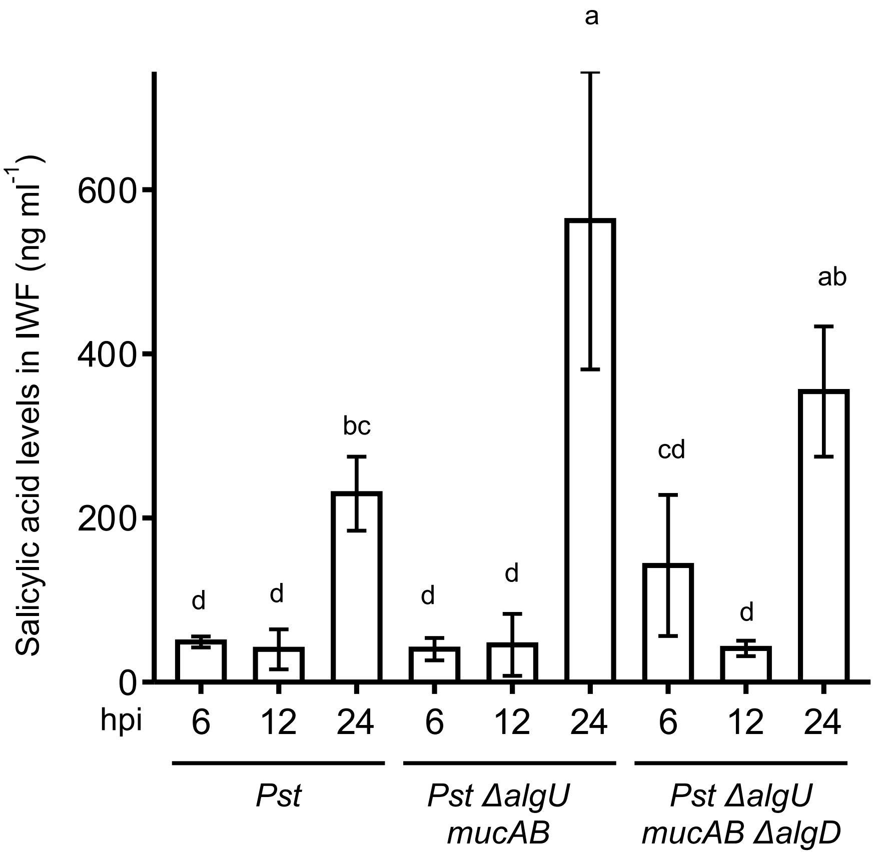

Supplement: Supplementary file 5 — Figure S5. Salicylic acid levels in intercellular washing fluids (IWFs) of Pseudomonas syringae pv. tomato (Pst)‐, Pst ΔalgU ΔmucAB‐, and Pst ΔalgU ΔmucAB ΔalgD‐inoculated leaves. [file MPP-25-e13403-s011.pdf]
