# Supplementary material for: Exploring Pseudomonas syringae pv. tomato biofilm‐like aggregate formation in susceptible and PTI‐responding Arabidopsis thaliana
Source: Mol Plant Pathol. 2023 Nov 21;25(1):e13403. doi: 10.1111/mpp.13403 (PMC10799205; doi:10.1111/mpp.13403)

Fig. S6 Quadratic correlation between bacterial levels and aggregation. Bacterial levels and aggregate formation were examined at 24-, 48-, and 72-hours post-inoculation with *Pst* using wild-type Col-0, the SA biosynthesis mutant *sid2-2*, and *fls2* (PTI-defective) mutants. Pooled data from these experiments was used in the correlation analysis, where the y-axis corresponds to the number of fields of view with aggregates divided by the total fields of view and the x-axis represents *in planta* bacterial levels (100 to 1 million cfu/ld). Bacterial levels and aggregation across 8 experiments, 3 plant genotypes, and 3 time points were used. $y = 0.4059 + 2.0257x + 0.4548x^2$. Adjusted R-squared = 0.42, $p = 2.129 \times 10^{-14}$, indicating that the model explains 42.0% of the variation around the mean, suggesting there is a correlation between aggregate formation and bacterial levels.

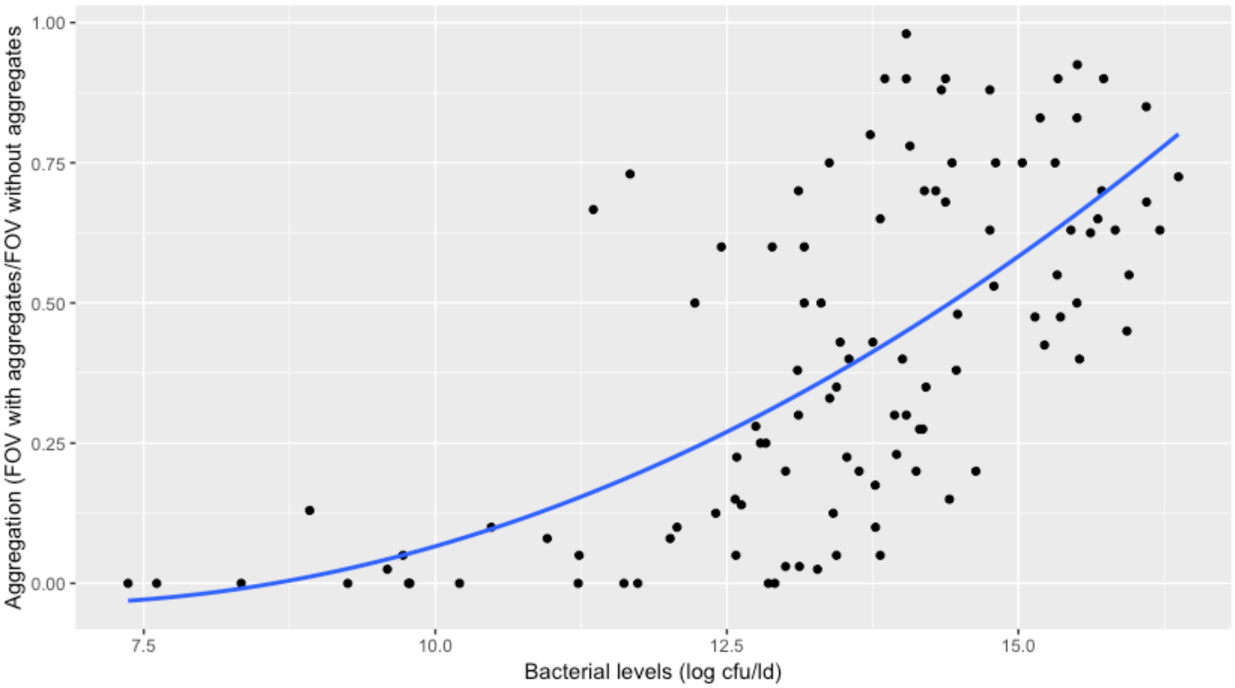

Supplement: Supplementary file 6 — Figure S6. Quadratic correlation between bacterial levels and aggregation. [file MPP-25-e13403-s010.pdf]
