# Supplementary material for: Exploring Pseudomonas syringae pv. tomato biofilm‐like aggregate formation in susceptible and PTI‐responding Arabidopsis thaliana
Source: Mol Plant Pathol. 2023 Nov 21;25(1):e13403. doi: 10.1111/mpp.13403 (PMC10799205; doi:10.1111/mpp.13403)

Fig. S7 Bacterial levels and biofilm-like aggregate formation during *Pst* infections *in planta*. Leaves were pressure-infiltrated with 1 μM flg22 (induced) or mock-treated with water. 24 hours later, the same leaves were inoculated with virulent GFP-expressing *Pst.* a) *In planta* bacterial quantitation of mock-treated and flg22-treated wild-type Col-0, *fls2* & *sid2-2* plants at 24hpi, 48hpi and 72 hpi, y axis-log scale. Asterisks indicate significant differences, $p < 0.05$ using the student's t-test. b) Aggregate formation was monitored by categorizing each microscopic field of view (40 FOV per treatment) as containing no bacteria, only planktonic bacteria, only bacterial aggregates, or both planktonic and bacterial aggregates at 24, 48, & 72 hpi. Letters indicate significant differences in aggregate size distribution, $p < 0.05$ using the Kruskal-Wallis test. c) Fluorescence microscopy images of biofilm-like aggregates observed at 72 hpi in leaves.

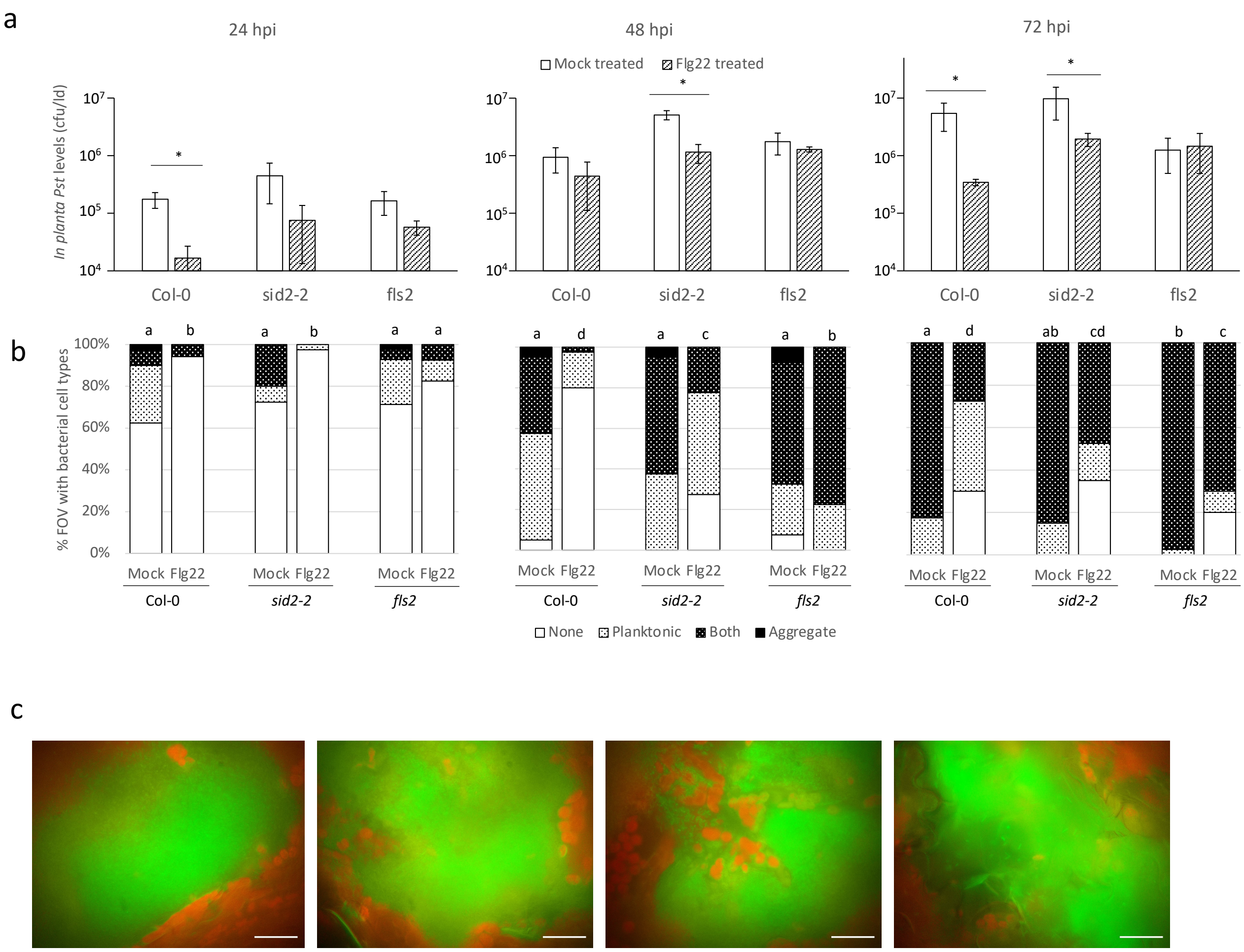

Supplement: Supplementary file 7 — Figure S7. Bacterial levels and biofilm‐like aggregate formation during Pseudomonas syringae pv. tomato infections in planta. [file MPP-25-e13403-s002.pdf]
