# Supplementary material for: Exploring Pseudomonas syringae pv. tomato biofilm‐like aggregate formation in susceptible and PTI‐responding Arabidopsis thaliana
Source: Mol Plant Pathol. 2023 Nov 21;25(1):e13403. doi: 10.1111/mpp.13403 (PMC10799205; doi:10.1111/mpp.13403)

Fig. S8. Bacterial multiplication during flg22-induced PTI

Leaves were pressure-infiltrated with 1 μM flg22 (induced) or mock-treated with water. 24 hours later, the same leaves were inoculated with virulent GFP-expressing *Pst. In planta* bacterial quantitation of mock-treated and flg22-treated wild-type Col-0 and *fls2* plants at 72 hpi, y axis-log scale. Asterisks indicate significant differences, $p < 0.05$ using the student's t-test. In this experiment, IWFs were collected at 6, 12, and 24 hours post inoculation (hpi). SA levels were quantified in IWFs using an SA biosensor assay (shown in Fig. 6).

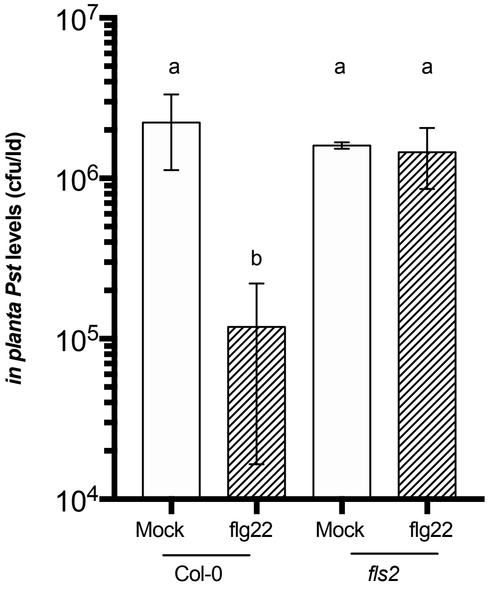

Supplement: Supplementary file 8 — Figure S8. Bacterial multiplication during flg22‐induced PAMP‐triggered immunity. [file MPP-25-e13403-s009.pdf]
