# Supplementary material for: Exploring Pseudomonas syringae pv. tomato biofilm‐like aggregate formation in susceptible and PTI‐responding Arabidopsis thaliana
Source: Mol Plant Pathol. 2023 Nov 21;25(1):e13403. doi: 10.1111/mpp.13403 (PMC10799205; doi:10.1111/mpp.13403)
Supplement: Supplementary file 9 — Method S1. Arabidopsis plant lines and growth conditions. METHOD S2 Bacterial strains and transformation. METHOD S3 PAMP‐triggered immunity assays and in planta quantification of bacterial levels. METHOD S4 Extracellular matrix staining and colocalization analysis. METHOD S5 Statistical analysis. METHOD S6 In vitro antibacterial growth curve assays. TABLE S1 Bacterial strains. [file MPP-25-e13403-s005.pdf]

Supplementary Experimental Methods

Method S1. *Arabidopsis* plant lines and growth conditions

Col-0, *sid2-2* (Nawrath & Métraux, 1999) and *fls2* (Kunze *et al.*, 2004) were used in these studies (*sid2-2* and *fls2* are in the Col-0 background). Seeds were surface-sterilized, stratified for 2 days in darkness at 4°C and then plated on Murashige and Skoog medium for germination under constant light. Approximately 1 week later, cotyledon-stage seedlings were transplanted to soil (Sunshine Mix #1) moistened with 1 g/l of all-purpose 20-20-20 fertilizer. Growth conditions were 22 ± 2°C, 80% ± 10% relative humidity, and 9 h of light (mixed fluorescent and incandescent, 110 to 130 µmol/m^2/s). Plants were grown to 3.5 to 4 weeks post germination (wpg) and then used in PTI assays.

Method S2. Bacterial strains and transformation

All strains of *P. syringae pv. tomato* DC3000 (*Pst*) including wild type, wild type carrying pDSK-GFPuv, and mutants of *Pst* were grown in King's Broth (KB) media (King *et al.*, 1954) overnight at room temperature (Table S1). *Pst* mutants, *Pst ΔalgD* (PS392) and *Pst ΔalgDΔalgUΔmucAB* (PS519), were created using wild-type *Pst* DC3000 (Markel *et al.*, 2016) and then were transformed with pDSK-GFPuv using a modified triparental mating method (Figurski & Helinski, 1979) in this work. *E. coli* DH5 α carrying the GFP plasmid, the helper strain *E. coli* RK600 and recipient strains of *Pst* (Table S1) were grown overnight separately and were centrifuged for 7 min at 1000g. Cell pellets were resuspended with 20 ml of 10 mM $MgCl_2$. Centrifugation and cell pellet resuspensions were repeated twice to remove any antibiotic residue from the overnight cultures. Each resuspended cell culture (50 µl each strain) was combined in 1 ml of KB media to make the mating mixture. After one hour of incubation at room temperature (22°C), 50 µl of the mating mixture was transferred onto a Luria-Bertani (LB) agar plate with rifampicin and kanamycin to select for successful transformed *Pst*. The successful transformation of *Pst* with GFP-expressing plasmid was confirmed by examining colonies under a UV lamp (365nm) to observe GFP florescence.

Method S3. PTI assays and *in planta* quantitation of bacterial levels

Overnight cultures of *Pst* were grown in KB media to mid-exponential phase (OD_{600}= 0.1-0.6) to ensure that all cells were alive in the inoculum (10^6 cfu/ml) and to ensure that similar numbers of bacterial cells were inoculated into leaves (~10^2 to 10^3 cells/leaf disc) at the start of the experiment (Kus et al, 2002, Carviel et

al., 2009, Carella et al., 2014). Cells were collected by centrifugation and resuspended in 10 mM $MgCl_2$ to 10^6 colony forming unit /ml (cfu/ml) and then inoculated by pressure-infiltration into fully expanded leaves using a needleless syringe. Prior to *Pst* inoculation, leaves were mock-treated with sterile dH_20 or PTI was induced by infiltrating leaves with flg22 (1μM, PhytoTech Labs #P6622). One day later, the same leaves were inoculated with 10^6 cfu/ml *Pst*, after which leaf bacterial levels were determined 3 days post inoculation. For quantification of *in planta* bacterial levels, three biological replicates of eight leaf disks (4 mm diameter) were collected and shaken at 200 rpm for 1 hour in 10 mM $MgCl_2$ with 0.1% Silwet L-77. Serial dilutions were plated on KB media with kanamycin (50 µg/ml) and rifampicin (100 µg/ml). Plates were incubated at room temperature for 2 days before determination of *Pst* levels (cfu/leaf disc (ld)). Fold differences between treatments were calculated in 2 steps. First, the average bacterial levels from 3 biological replicates were calculated for each treatment group. Second, the mock-treated average group was divided by the flg22-treated group average to give the fold difference in bacterial levels between the 2 treatments.

Method S4. Extracellular matrix staining and co-localization analysis

Plants were inoculated with 10^6 cfu/ml *Pst* pDSK-GFPuv (Wang *et al.*, 2007). At 48 hours, leaves were cut at the petiole and sections of the lower epidermis were removed using tape. Sections without the lower epidermis were isolated using a razor blade and were mounted on a glass slide with the epidermis-less surface facing upwards. Different leaves were stained to detect eDNA or extracellular polysaccharides.

Extracellular DNA Detection

A DAPI working solution (0.1 µg/ml) was made fresh from a DAPI stock solution (1mg/ml) (Sigma-Aldrich, CAS #: 28718-90-3) that was stored at -20 °C. DAPI working solution was added to cover the entire leaf sample on the slide (15-30 µl per sample) and samples were incubated in the dark for 5 mins. After incubation, the staining solution was removed and 50µl of sterile deionised water was gently pipetted onto the samples to rinse them. The rinsing step was repeated three times. After removing excess liquid, a cover slip was gently placed on each slide. A UV filter (435-485 nm) was used during florescence microscopy with the Zeiss Axioscope epifluorescence microscope fitted with the Zeiss AxioCam ICm 1 monochrome camera and a C-Mount lens.

Extracellular Polysaccharide Detection

Concanavalin A-Tetramethylrhodamine Conjugate (ConA-TRIC, Invitrogen™ by ThermoFisher, Cat #: C860) stock solution (2 µg/µl) was made ahead of time and stored at -20 °C, then diluted to make a fresh solution at the working concentration (0.4 µg/µl) with PBS buffer. To stain α-polysaccharides, ConA-TRIC working solution was added to cover the entire leaf sample on the slide (15-30 µl per sample) and samples were incubated in the dark for 30 mins. After incubation, the staining solution was removed and 50 µl of PBS buffer was gently pipetted onto the samples to rinse them one time. After ConA-TRIC staining, the Calcofluor White (CFW stock solution, Sigma-Aldrich, Cat# 18909-100ML-F) working dye solution was prepared freshly by mixing the CFW stock with 10% KOH solution in a ratio of 3:2. The CFW working dye solution was pipetted (15-30 µl per sample) to cover the samples to stain β-polysaccharides. Slides were incubated in the dark for 5 mins at 22°C. After incubation, the dye solution was removed, and sterile water was pipetted onto the slide to rinse the samples. The excess liquid was removed, and a cover slip was gently placed on each slide. A Cy5 filter (663-738 nm) was used to observe ConA-TRIC signals, and a UV filter (435-485 nm) was used observe CFW signals during florescence microscopy using the Zeiss Axioscope epifluorescence microscope.

Co-localization of *Pst*-GFP and ConA-TRIC and CFW signals

To quantitatively determine if GFP-expressing bacteria co-localized with regions of ConA-TRITC- or calcofluor white-assocciated signals, GFP fluorescence images were superimposed with the corresponding ConA-TRITC or calcofluor white stained images. ImageJ (Abramoff *et al.,* 2004) with the JACoP plugin (Bolte & Cordelières, 2006) was used to calculate colocalization of GFP signals with either ConA-TRITC or calcofluor white signals. Pearson's correlation coefficient represents the correlation of a scatterplot where pixel intensity of a single pixel in one channel is used as the x-coordinate (ie. GFP) and the pixel intensity of the same pixel in another channel (ie. ConA-TRITC or CFW) is used as the y-coordinate. This process is repeated for every pixel in the image and Pearson's correlation is then calculated from the resulting scatterplot. Li's intensity correlation analysis calculates the product of $(A_i$-a)$(B_i$-b) where A_i represents the pixel intensity of the current pixel on the GFP channel, a represents the mean pixel intensity of the GFP channel, B_i represents the pixel intensity of the current pixel on the stain (ie. ConA-TRITC or CFW) channel, and b represents the mean pixel intensity of the stain channel. This value is then calculated for every pixel of the merged image and Li's intensity correlation quotient is defined as the ratio of pixels that generated a

positive value to the total number of pixels analyzed subtracted by 0.5 (Li *et al.,* 2004). For Mander's split co-occurance, for each image a pixel intensity threshold was manually set for all channels and Mander's split co-occurance is defined as the number of pixels above the specified threshold in both channels (overlapping flourescence) divided by the the total number of pixels above the threshold of a single channel.

Method S5. Statistical Analysis

Statistical significance was determined using a Student's t-test or an analysis of variance (ANOVA), or a Kruskal-Wallis test as indicated. For the Student's t-test, a two-tailed test for either equal or unequal variance was performed ($p < 0.05$). Single variable ANOVA analysis was performed with ($p < 0.05$) and followed up with a post-HOC test, Tukey's HSD. The Kruskal-Wallis test, a non-parametric statistical method, was conducted to assess potential significant differences in aggregate size distribution among multiple groups ($p < 0.05$). Subsequently, a Dunn's test was employed to identify specific group pairs that exhibited statistically significant variations.

Method S6. *In vitro* antibacterial growth curve assays

Antibacterial assays with pure SA were performed as described previously (Wilson *et al.*, 2017). Briefly, overnight cultures of *Pst* strains were grown in KB media. Cells were centrifuged and the pellets washed two times with hrp-inducing minimal media (HIM) (50 mM potassium phosphate, 10 mM D-fructose, 7.6 mM $(NH_4)2SO2$, 6.8 mM $MgCl_2$, 1.7 mM NaCl, pH 5.7) or KB media. Bacterial cultures were diluted to an optical density (OD_{600}) of 0.1. Aliquots of 160 µl of HIM- or KB-diluted bacterial culture were added to each well of a 96-well plate. SA was first serial diluted in anhydrous ethanol to concentrations that ranged 250 to 0.5 mM. 3.2 µl aliquots of the serial dilutions were added to 160 µl of bacterial solution to reach the indicated final SA concentration and a final ethanol concentration of 2.0%. The 96-well plate was incubated with shaking at 26^0C for 72 hours. Optical density at 600nm was measured every 15 mins using a Tecan Sunrise plate reader. After 72 hours, 10 µl aliquots of the cell suspension were spotted onto a KB agar plate and observed two days later to test for bactericidal activity.

Table S1. Bacterial Strains

Genotype	Transformed Plasmid	Reference
P. syringae pv. *tomato* DC3000 (*Pst* wild-type)		
Pst wild-type	pDSK-GFPuv	Whalen et al., 1991 & Wang et al., 2007
Pst ΔalgD	pDSK-GFPuv	Markel et al., 2016, this work
Pst ΔalgDΔalgUΔmucAB	pDSK-GFPuv	Markel et al., 2016, this work
E. coli		
E. Coli DH5α	*	Figurski & Helinski, 1979
E. Coli RK600	pRK600	Figurski & Helinski, 1979

*not transformed with any plasmids
