# Supplementary material for: Exploring Pseudomonas syringae pv. tomato biofilm‐like aggregate formation in susceptible and PTI‐responding Arabidopsis thaliana
Source: Mol Plant Pathol. 2023 Nov 21;25(1):e13403. doi: 10.1111/mpp.13403 (PMC10799205; doi:10.1111/mpp.13403)
Supplement: Supplementary file 10 — Table S2. The PAMP‐triggered immunity response varies across experiments. [file MPP-25-e13403-s004.pdf]

Table S2. The PTI response varies across experiments

Genotype	PTI Response in Eight Experiments[1,2]							
	1	2	3	4	5[4]	6	7	8
Col-0	32	23	100	52	68	9	16	8
fls2	Nd[3]	nd	nd	nd	nd	nd	nd	nd
sid2-2	nd	7	16	7	5	nd	5	7

[1] Leaves were infiltrated with water (mock-treated) or 1 μM flg22 (flg22-treated), 24 hours later, the same leaves were inoculated with virulent GFP-expressing *Pst. In planta* bacterial levels were determined at 72 hpi.

[2]The PTI response was calculated as the fold difference in *Pst* levels in leaves that were mock-treated versus flg22-treated and statistically significant fold differences were determined using a two-way ANOVA (Tukey's HSD, $P<0.05$).

[3] nd = no difference in *Pst* levels between flg22-treated and mock-treated leaves.

[4] Experiment 5 is displayed in Figure 2A
