# Supplementary material for: Exploring Pseudomonas syringae pv. tomato biofilm‐like aggregate formation in susceptible and PTI‐responding Arabidopsis thaliana
Source: Mol Plant Pathol. 2023 Nov 21;25(1):e13403. doi: 10.1111/mpp.13403 (PMC10799205; doi:10.1111/mpp.13403)
Supplement: Supplementary file 11 — Table S3. Percentage of fields of view (FOVs) with Pseudomonas syringae pv. tomato aggregates in flg22‐ and mock‐treated leaves. [file MPP-25-e13403-s008.pdf]

Table S3. Percent Fields of View (FOV) with *Pst* aggregates in flg22- and mock-treated leaves

		% FOV with *Pst* aggregates in leaves[2]							
		Experiment[3]							
**Genotype**	**Treatment[1]**	**1**	**2**	**3**	**4**	**5**	**6**	**7**	**8**
Col-0	Mock	75	80	40	60	40	40	20	55
	flg22	0	25	0	0	5	3	0	10
sid2-2	Mock	90	90	90	60	70	60	20	50
	flg22	25	40	50	20	45	20	20	5
fls2	Mock	90	90	70	75	50	70	30	40
	flg22	75	90	30	80	90	80	30	45

[1] Leaves of Col-0, *sid2-2*, and *fls2* were treated with 1 μM flg22 (flg22-treated) or mock-treated with water. 24 hours later, the same leaves were inoculated with virulent GFP-expressing *Pst.*

[2] FOV with *Pst* aggregates was determined by categorizing each microscopic field of view as with or without aggregates and calculating the percentage of FOV with aggregates.

[3] Table S2 and Table S3 display data for the same 8 experiments.
