# Supplementary material for: Exploring Pseudomonas syringae pv. tomato biofilm‐like aggregate formation in susceptible and PTI‐responding Arabidopsis thaliana
Source: Mol Plant Pathol. 2023 Nov 21;25(1):e13403. doi: 10.1111/mpp.13403 (PMC10799205; doi:10.1111/mpp.13403)
Supplement: Supplementary file 12 — Table S4. PAMP‐triggered immunity responses across seasons. [file MPP-25-e13403-s013.pdf]

Table S4.PTI responses across seasons

2018-2019	Level of resistance in flg22-treated Col-0 plants = fold decrease in *Pst* levels in flg22- vs mock-treated plants	Corresponding experiment number in Table S1[1]
Spring (Mar-May)	23, 100, 52	2, 3, 4
Summer (Jun-Aug)	68, 9, 16	5, 6, 7
Fall (Sep-Nov)	8	8
Winter (Dec-Feb)	32	1

[1] See Table S2 for more details
