# Supplementary material for: Exploring Pseudomonas syringae pv. tomato biofilm‐like aggregate formation in susceptible and PTI‐responding Arabidopsis thaliana
Source: Mol Plant Pathol. 2023 Nov 21;25(1):e13403. doi: 10.1111/mpp.13403 (PMC10799205; doi:10.1111/mpp.13403)
Supplement: Supplementary file 13 — Table S5. Effect of salicylic acid on Pseudomonas syringae pv. tomato (Pst) GFP and Pst mutants cultured in apoplast‐mimicking medium (HIM**). [file MPP-25-e13403-s003.pdf]

Table S5. Effect of salicylic acid on *Pst* GFP & *Pst* mutants cultured in apoplast mimicking media (HIM).**

Strain	Salicylic acid MIC[†] (Frequency[*])	MBC[‡] (Frequency[*])
Pst GFP	1 mM (1/3)<br>2 mM (2/3)	2 mM (3/8)<br>5 mM (5/8)
Pst ΔalgD	1 mM (2/4)<br>2 mM (2/4)	2 mM (2/6)<br>5 mM (4/6)
Pst ΔalgD ΔmucAB ΔalgU	0.5 mM (1/5)<br>1 mM (4/5)	1 mM (1/6)<br>2 mM (2/6)<br>5 mM (3/6)

† Minimum inhibitory concentration (MIC) corresponds to lowest concentration that completely inhibited growth

‡ Minimum bactericidal concentration (MBC) corresponds to lowest concentration at which 0 colonies were observed after cultures were transferred to solid KB media

* # of occurrences / total # of experiments

** HIM = Hrp-inducing minimal media
